# Oral polyphenol-armored nanomedicine for targeted modulation of gut microbiota–brain interactions in colitis

**DOI:** 10.1126/sciadv.adf3887

**Published:** 2023-05-26

**Authors:** Huan He, Qiaozhen Qin, Fang Xu, Yitong Chen, Shuquan Rao, Chao Wang, Xiaoxia Jiang, Xiong Lu, Chaoming Xie

**Affiliations:** ^1^ Institute of Biomedical Engineering, College of Medicine, Southwest Jiaotong University, Chengdu 610031, Sichuan, China.; ^2^ Key Laboratory of Advanced Technologies of Materials Ministry of Education, School of Materials Science and Engineering, Southwest Jiaotong University, Chengdu 610031, China.; ^3^ Beijing Institute of Basic Medical Sciences, Beijing, 100850, China.; ^4^ Institute of Functional Nano and Soft Materials (FUNSOM), Jiangsu Key Laboratory for Carbon-based Functional Materials and Devices, Soochow University, Suzhou, Jiangsu 215123, China.; ^5^ State Key Laboratory of Experimental Hematology, National Clinical Research Center for Blood Diseases, Haihe Laboratory of Cell Ecosystem, Institute of Hematology & Blood Diseases Hospital, Chinese Academy of Medical Sciences & Peking Union Medical College, Tianjin, 300020, China.

## Abstract

Developing oral nanomedicines that suppress intestinal inflammation while modulating gut microbiota and brain interactions is essential for effectively treating inflammatory bowel disease. Here, we report an oral polyphenol-armored nanomedicine based on tumor necrosis factor–α (TNF-α)–small interfering RNA and gallic acid–mediated graphene quantum dot (GAGQD)–encapsulated bovine serum albumin nanoparticle, with a chitosan and tannin acid (CHI/TA) multilayer. Referred to “armor,” the CHI/TA multilayer resists the harsh environment of the gastrointestinal tract and adheres to inflamed colon sites in a targeted manner. TA provides antioxidative stress and prebiotic activities that modulate the diverse gut microbiota. Moreover, GAGQD protected TNF-α–siRNA delivery. Unexpectedly, the armored nanomedicine suppressed hyperactive immune responses and modulated bacterial gut microbiota homeostasis in a mouse model of acute colitis. Notably, the armored nanomedicine alleviated anxiety- and depression-like behaviors and cognitive impairment in mice with colitis. This armor strategy sheds light on the effect of oral nanomedicines on bacterial gut microbiome-brain interactions.

## Teaser

An oral polyphenol-armored nanomedicine alleviates cognitive dysfunctions through modulating gut-brain axis.

## INTRODUCTION

Inflammatory bowel disease (IBD), primarily Crohn’s disease and ulcerative colon inflammation, is an idiopathic disease with relapsing, refractory, and uncontrolled inflammatory responses in the ileum, rectum, and colon (*1*, *2*). Accumulating evidence supports oxidative stress and gut microbial disturbances as crucial triggers for IBD (*3*, *4*). Oxidative stress causes neutrophils and macrophages to infiltrate the intestinal mucosa at the site of IBD, where they release large amounts of reactive oxygen species (ROS) and inflammatory cytokines (*5*). Reducing inflammatory cytokines and scavenging ROS are effective strategies for relieving IBD (*6*–*8*). Treatment of IBD by inhibiting inflammatory factors overexpressed at the site of inflammation through the delivery of small interfering RNA (siRNA) nanomedicines, such as tumor necrosis factor–α siRNA (TNF-α–siRNA), has become popular in recent years (*9*). The general method of intravenous injection of siRNA nanomedicines is associated with high risks, and systemic blood delivery results in insignificant drug concentrations at the target site. Although enemas can achieve higher drug concentrations in the colon, they can also disrupt patient compliance and cause distress.

Oral administration is a convenient, safe, and desirable route for treating IBD (*10*, *11*). However, IBD-induced diarrhea can lead to rapid drug clearance, whereas digestive fluids lead to rapid degradation of the delivery system (*12*). Although various delivery systems have been developed, insufficient residence time, low targeting efficiency for inflamed colons, and complex synthetic procedures limit their clinical applications (*13*). High extracellular and intracellular ROS levels in the inflammatory environment can deactivate siRNAs (*14*). Therefore, an effective siRNA delivery system that prevents gastrointestinal degradation, provides targeted adhesion, and achieves long-term retention in the inflamed colon is essential for improving the efficiency of colitis treatment.

Gut microbiota affects brain physiology, including behavioral and cognitive functions (*15*, *16*). Although traditional medical treatments for IBD have primarily focused on suppressing intestinal immune responses, they generally do not address the underlying causes of IBD, such as dysbiosis of the gut microbiota and physiological dysfunction of the brain associated with behavior and cognition (*17*–*19*). The gut-brain axis regulates bidirectional communication between the brain and the gastrointestinal tract (*20*). Gut inflammation, gut microbiota dysbiosis, and changes in gut barrier structure and function can lead to neuroinflammation and other emotional and behavioral disorders through three mechanisms: (i) signals directly transmitted from the gut to the brain through a neural network, (ii) inflammatory factors from immune cells in the gut that regulate the brain, and/or (iii) metabolites produced by gut microbes that enter the bloodstream and brain to affect behavior (*21*, *22*). Polyphenols, such as tannins, are a diverse class of secondary plant metabolites available in food with antioxidant and ROS-scavenging activities (*23*, *24*). Polyphenols have poor adsorption rates in the small intestine, and unabsorbed polyphenols can be catabolized by microbiota into more active microbial metabolites in the colon (*25*, *26*). Consequently, polyphenols exhibit prebiotic activities that increase the relative abundance of beneficial bacteria, thereby promoting the maintenance of gut microbiota homeostasis (*27*). Thus, designing a polyphenol-based nanomedicine that can inhibit the inflammatory response, improve intestinal barrier function, reshape the intestinal microbiome, and relieve cognitive dysfunction is beneficial for thoroughly treating patients with IBD.

In this study, we propose a polyphenol-armored strategy to develop an oral nanomedicine with anti-oxidative stress, anti-digestive, and inflammation-targeted activities. Specifically, we developed a polyphenol-armored nanomedicine composed of TNF-α–siRNA, a gallic acid–mediated graphene quantum dot (GAGQD)–encapsulated bovine serum albumin (BSA) nanoparticle (siRNA-GBSA NP) core, and chitosan and tannin acid (CHI/TA)*_n_* multilayer shell. TA, a food-grade plant polyphenol, resists the extreme environment of the stomach. TA is rich in phenolic hydroxyl groups, which can endow NPs with bioadhesive and antioxidant properties. Moreover, TA can modulate microbiota and alleviate brain diseases (*28*, *29*). The CHI/TA multilayer “armor” was constructed on the surface of the nanomedicine via layer-by-layer (LbL) self-assembly. With this armor, NPs could successfully pass through the stomach and small intestine, consequently targeting and accumulating on inflamed colon sites. They exhibited long-term residence after oral administration in a mouse model of dextran sulfate sodium (DSS)–induced acute colitis. In addition, armor can regulate bacterial gut microbial homeostasis to restore brain cognition. Last, GAGQD has a high ROS-scavenging ability, which protects siRNA during delivery to suppress intestinal immune responses effectively.

## RESULTS

### Design strategy

**Fig. 1.**
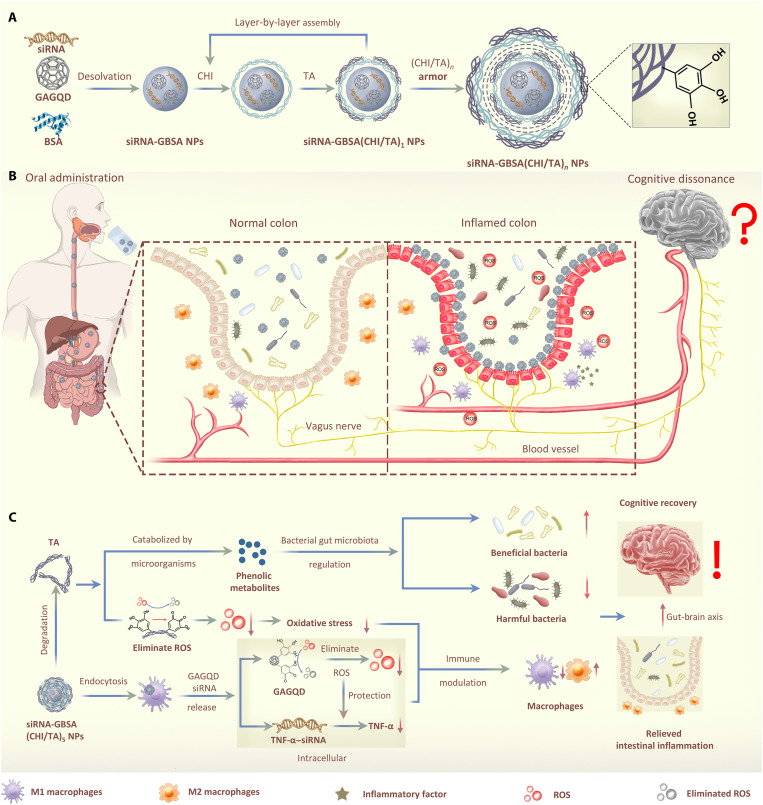
Polyphenol-armored nanomedicine and how it modulates gut microbiota–brain interactions in colitis. (**A**) Synthetic scheme of siRNA-GBSA(CHI/TA)*_n_* NPs. (**B**) siRNA-GBSA(CHI/TA)_5_ NPs were orally administered by gavage and passed smoothly through the extreme stomach environment under the protection of (CHI/TA)_5_ armor. The armor slowly degraded at the intestinal site, enriching the inflamed colon through electrostatic adsorption and polyphenol adhesion. (**C**) The microenvironment of the inflamed colon site was regulated by relieving the inflammatory responses and alleviating the imbalance of intestinal flora by siRNA-GBSA(CHI/TA)_5_ NPs. Moreover, inflammation in the brains of IBD mice was also alleviated via the gut-brain axis and accompanied by decreased activation of astrocytes, microglia, and neuroinflammatory cells in the brain. Consequently, the relief of brain inflammation improved cognitive performance.

GAGQD was prepared by a hydrothermal method using citric acid and gallic acid (GA) as carbon sources and ethylenediamine as the nitrogen source (Fig. 1A) (*30*). Next, TNF-α–siRNA and GAGQD were encapsulated in BSA using a desolvation method to form negatively charged siRNA-GBSA NPs. To enable siRNA-GBSA NPs to pass through the harsh environment of the gastrointestinal tract and accumulate at inflamed colon sites in a targeted manner, a CHI/TA multilayer armor was prepared on the surface of siRNA-GBSA NPs via electrostatic LbL self-assembly (Fig. 1A). The number of layers of (CHI/TA)*_n_* can be adjusted to avoid rapid degradation in the gastrointestinal tract. The CHI/TA armor enabled the NPs to smoothly pass through the stomach and maintain their morphological integrity in the gastric fluid (Fig. 1B). Moreover, the CHI/TA armor slowly degrades after entering the small intestine. After reaching the colonic site, negatively charged NPs targeted the positively charged inflamed colonic sites. At the same time, the phenolic hydroxyl groups of TA in the CHI/TA armor further endowed NPs with ROS-scavenging activity and tissue adhesion, prolonging their residence time in the colon. Owing to the long-term residence time and cell affinity of TA, NPs are highly susceptible to endocytosis by macrophages. Following intracellular enzymatic degradation, TNF-α–siRNA was released from BSA NPs protected by GAGQD, ultimately eliminating inflammation. In addition, after the degradation of the armor, TA was catabolized by the gut microbiota into more active phenolic metabolites capable of regulating gut microbiota–brain interactions. Thus, armored nanomedicine suppressed intestinal immune responses and regulated bacterial gut microbiota homeostasis, consequently relieving brain inflammation and improving the behavioral and cognitive recovery of IBD model mice.

### Characterization of polyphenol-armored nanomedicine

**Fig. 2.**
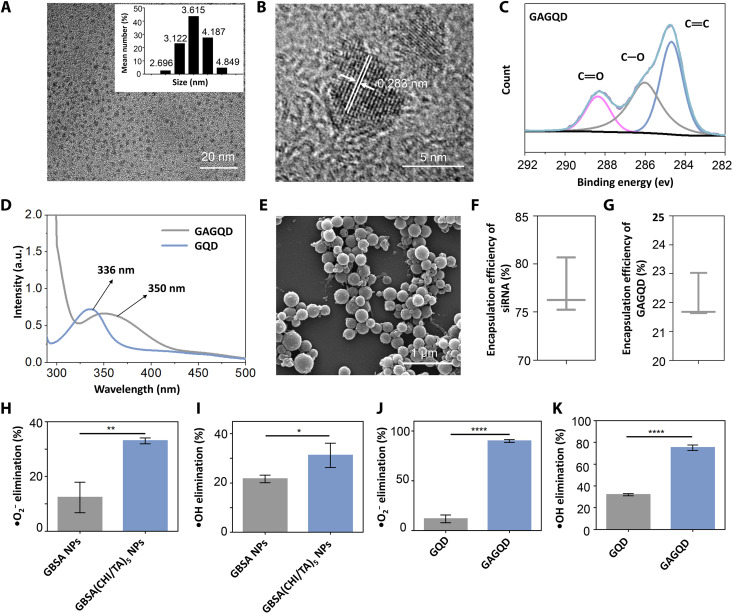
Characterizations of the polyphenol-armored nanomedicine. (**A**) TEM images and particle size distribution maps of GAGQD. (**B**) HRTEM of GAGQD. (**C**) High-resolution x-ray photoelectron spectroscopy data of C1s for GAGQD. (**D**) UV-vis absorption spectra of GQD and GAGQD. a.u., arbitrary units. (**E**) Scanning electron microscopy (SEM) images of siRNA-GBSA(CHI/TA)_5_ NPs. (**F** and **G**) Encapsulation efficiency of siRNA or GAGQD in siRNA-GBSA NPs. (**H** and **I**) •O_2_^−^ elimination activity and •OH elimination activity of GBSA NPs and GBSA(CHI/TA)_5_ NPs. (**J** and **K**) •O_2_^−^ elimination activity and •OH elimination activity of GQD and GAGQD. (Data are presented as the mean ± SD; *P < 0.05, **P < 0.01, and ****P < 0.0001).

Transmission electron microscopy (TEM) images showed that the GAGQD are monodisperse spherical particles with a size distribution of 2 to 5 nm (Fig. 2A). High-resolution TEM images of GAGQD showed an interlayer spacing of 0.28 nm, corresponding to the (100) crystal plane of graphitic carbon (Fig. 2B) (*31*). X-ray photoelectron spectroscopy analysis revealed characteristic peaks of C─O and C═O bonds at 285 and 288 eV, corresponding to phenolic hydroxyl and quinone groups in the GAGQD, respectively. The C─O bonds of GAGQD are notably stronger than those of GQD, illustrating the enhanced abundance of phenolic hydroxyl groups in GAGQD with the introduction of GA (Fig. 2C and fig. S1). The Fourier-transform infrared spectroscopy (FTIR) results were consistent (fig. S2) (*32*). The ultraviolet-visible (UV-vis) absorption spectra of the GQD and GAGQD showed intense characteristic absorption peaks at 336 and 350 nm, respectively (Fig. 2D), attributed to the *n*-π* transition of the C═O bond. The red-shift phenomenon of GAGQD may be attributed to the conjugation effect caused by the introduction of phenolic hydroxyl auxochrome groups (*33*). The fluorescence spectra of the GAGQD showed the most intense emission at 510 nm with 400 nm excitation (fig. S3). The Commission Internationale d’Eclairage coordinates of GAGQD are located in the green region of the visible spectra (fig. S4).

After encapsulation and LbL assembly, siRNA-GBSA NPs, siRNA-GBSA(CHI/TA)_2_ NPs, and siRNA-GBSA(CHI/TA)_5_ NPs displayed spherical structures (Fig. 2E and fig. S5). The sizes of siRNA-GBSA NPs, siRNA-GBSA(CHI/TA)_2_ NPs, and siRNA-GBSA(CHI/TA)_5_ NPs were approximately 200, 230, and 350 nm, respectively (fig. S6). The switching of positive and negative surface charges and increased size of siRNA-GBSA(CHI/TA)*_n_* NPs indicated the successful LbL self-assembly of positive CHI and negative TA (fig. S7). The encapsulation efficiencies of siRNA and GAGQD in the BSA NPs were 77.3 and 22.1%, respectively (Fig. 2, F and G).

### ROS scavenging activity

The scavenging activities of GQD, GAGQD, GBSA NPs, and GBSA(CHI/TA)_5_ NPs against •O_2_^−^, •OH (representative ROS in IBD), and H_2_O_2_ were investigated (Fig. 2, H to K, and fig. S8). The ROS-scavenging and catalase (CAT) activities of the GBSA NPs were attributed to the partially exposed GAGQDs. In contrast, the higher ROS-scavenging and CAT activities of GBSA(CHI/TA)_5_ NPs were attributed to the abundant phenolic hydroxyl groups of TA on the armor, which can protect cells from damage by ROS and H_2_O_2_ in IBD (Fig. 2, H and I). Moreover, after cellular uptake, the delivered GAGQD protected the siRNA from damage by intracellular ROS. As shown in Fig. 2 (J and K) and fig. S8, GAGQD have higher CAT activity and clearance efficiencies for •O_2_^−^ and •OH than GQD, demonstrating excellent ROS-scavenging activity. The excellent ROS-scavenging and CAT activities of GAGQD were attributed to the phenolic hydroxyl groups of GA.

**Fig. 3.**
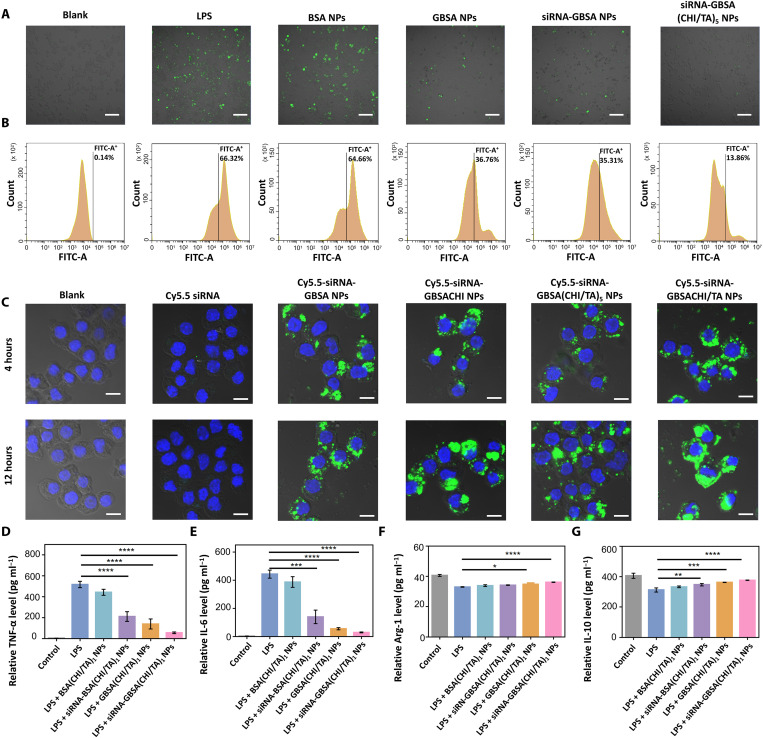
ROS scavenging activity of the polyphenol-armored nanomedicine. (**A**) Intracellular ROS staining images and (**B**) flow cytometry analysis of DCFH-labeled RAW264.7. Cells were subjected to different treatments. Scale bars, 100 μm. (**C**) Confocal images of RAW264.7 cells after incubation with 200 μg ml^−1^ of different NPs for 4 or 12 hours. Scale bars, 10 μm. (**D** to **G**) Enzyme-linked immunosorbent assay (ELISA) results showed extracellular expression of (D) TNF-α, (E) IL-6, (F) Arg-1, and (G) IL-10. (Data are presented as the mean ± SD; *P < 0.05, **P < 0.01, ***P < 0.001, and ****P < 0.0001).

To investigate whether siRNA-GBSA(CHI/TA)*_n_* NPs (*n* = 0 to 5) could protect cells from ROS-induced damage, a cellular inflammation model was established by treating RAW264.7 macrophages with lipopolysaccharide (LPS). ROS levels were analyzed by confocal laser scanning microscopy (CLSM) and flow cytometry using a 2',7'-Dichlorodihydrofluorescein diacetate (DCFH-DA) probe (Fig. 3, A and B). LPS-induced macrophages (LPS group) displayed the most intense fluorescence (66.32%), which was not notably reduced after BSA NP treatment (64.66%). Owing to the partial exposure to GAGQD, the fluorescence intensities of the GBSA NPs and siRNA-GBSA NP groups were reduced to 36.76 and 35.31%, respectively. Moreover, with armor, the fluorescence intensity of siRNA-GBSA (CHI/TA)_5_ NPs was substantially reduced to 13.86%, indicating excellent ROS-scavenging activity that protected cells against ROS-induced damage. The siRNA-GBSA(CHI/TA)*_n_* NPs were noncytotoxic in the concentration range of 0 to 600 μg ml^−1^ (fig. S9).

### Cellular uptake of polyphenol-armored nanomedicine in vitro

Effective intracellular siRNA delivery is essential to transfection effectiveness (*34*). Cellular uptake of polyphenol-armored nanomedicines encapsulating Cy5.5-labeled siRNA (Cy5.5-siRNA) was confirmed using CLSM (Fig. 3C). As expected, prominent green fluorescence was observed with the delivery of Cy5.5-siRNA-GBSA, Cy5.5-siRNA-GBSACHI, Cy5.5-siRNA-GBSA(CHI/TA)_5_, and Cy5.5-siRNA-GBSA(CHI/TA)_1_ NPs, demonstrating that these NPs can be endocytosed by cells. The Cy5.5-siRNA group essentially had no green fluorescence when incubated for 4 or 12 hours, indicating that free siRNA hardly entered the cells. The endocytosis efficiency of the NPs was further quantified by flow cytometry (fig. S10). The rate of NP endocytosis increased with an increase in residence time, indicating the importance of prolonged NP residence at the target site for improved endocytosis efficiency. Cy5.5-siRNA-GBSA(CHI/TA)_5_ NPs showed the highest rate of endocytosis at 4 hours compared to Cy5.5-siRNA-GBSA NPs and Cy5.5-siRNA-GBSACHI NPs, indicating that TA modification improved the cell affinity of the nanomedicine. In addition, Cy5.5-siRNA-GBSA(CHI/TA)_5_ NPs were found to be degraded in the colon. Therefore, we used Cy5.5-siRNA-GBSA(CHI/TA)_1_ NPs with a single protective layer of CHI/TA to mimic NPs in the colon after partial degradation. The results showed a higher rate of endocytosis of Cy5.5-siRNA-GBSA(CHI/TA)_1_ NPs compared with Cy5.5-siRNA-GBSA(CHI/TA)_5_ NPs, which the smaller size of the single-layer NPs may cause. Thus, we conclude that efficient endocytosis of siRNA-GBSA(CHI/TA)_5_ NPs can be achieved in the colon, even if the (CHI/TA)_5_ armor is partially degraded in the gastrointestinal tract.

### In vitro anti-inflammatory activity

The anti-inflammatory capacity of siRNA-GBSA(CHI/TA)_1_ NPs in vitro was estimated by evaluating cytokine expression in LPS-stimulated RAW264.7 cells. The results showed that GBSA(CHI/TA)_1_ NPs and siRNA-BSA(CHI/TA)_1_ NPs significantly inhibited the expression of TNF-α and interleukin-6 (IL-6) (Fig. 3, D and E). Moreover, siRNA-GBSA(CHI/TA)_1_ NPs displayed the highest anti-inflammatory ability, attributed to the synergy of the ROS-scavenging ability of GAGQD, gene-silencing efficiency of TNF-α siRNA, and efficient intracellular endocytosis. In addition, the relative expression levels of the M2 macrophage–related genes IL-10 and arginase-1 (Arg-1) were evaluated. The results showed that siRNA-GBSA(CHI/TA)_1_ NPs significantly up-regulated the expression of IL-10 and Arg-1 (Fig. 3, F and G), indicating that siRNA-GBSA(CHI/TA)_1_ NPs exerted anti-inflammatory effects by suppressing M1 macrophage polarization and promoting M2 macrophage activation.

### Extreme environment resistance and long-term retention

**Fig. 4.**
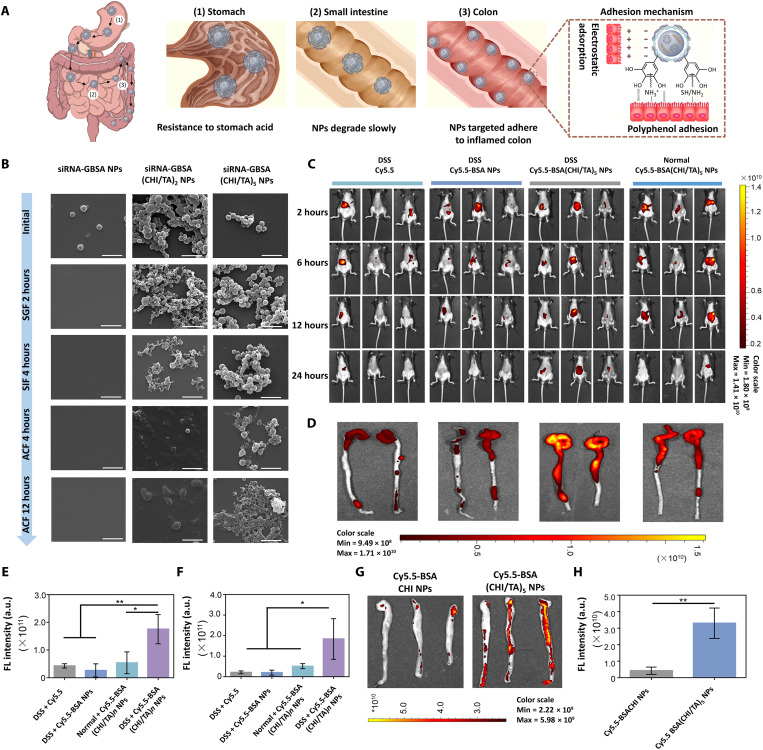
Long-term retention activity of the polyphenol-armored nanomedicine in the inflamed colon. (**A**) Diagram showing how siRNA-GBSA(CHI/TA)_5_ NPs resist degradation in the gastrointestinal tract and eventually accumulate in the colon. (**B**) SEM images of siRNA-GBSA NPs, siRNA-GBSA(CHI/TA)_2_ NPs, and siRNA-GBSA(CHI/TA)_5_ NPs after incubation in simulated gastric fluid (SGF) for 2 hours, simulated intestinal fluid (SIF) for 4 hours, and simulated colonic fluid (SCF) for 4 and 12 hours. Scale bars, 1 μm. (**C**) In vivo small animal fluorescence imaging of normal and IBD mice (drinking 3% DSS sterile water for 3 days) at different time points (3, 6, 12, and 24 hours) after intragastric administration of Cy5.5, Cy5.5-BSA NPs, and Cy5.5-BSA(CHI/TA)_5_ NPs. (**D**) In vitro fluorescence images of colons after gavage of Cy5.5, Cy5.5-BSA NPs, or Cy5.5-BSA(CHI/TA)_5_ NPs for 24 hours. (**E** and **F**) Corresponding fluorescence (FL) quantitative statistics of in vivo fluorescence imaging of mice and in vitro fluorescence of colon after 24 hours of intragastric administration. (**G**) In vitro fluorescence images and (**H**) fluorescence quantitative analysis of mouse colons cultured with Cy5.5-BSACHI NPs or Cy5.5-BSA(CHI/TA)_5_ NPs for 1 hour, following three washes. (Data are presented as the mean ± SD; *P < 0.05 and **P < 0.01).

Resistance to the extreme environment of the gastrointestinal tract and sufficient long-term retention in the colon are crucial features of orally administered nanomedicines for treating colitis. Armored siRNA-GBSA(CHI/TA)_5_ NPs effectively resisted the gastric and intestinal fluids to reach the colon successfully. Moreover, the negative surface charge of the CHI/TA multilayer allows NPs to target and adhere to inflamed sites in the colon, which have positively charged proteins such as transferrin and antimicrobial peptides (*35*). Furthermore, the phenolic hydroxyl groups on the CHI/TA armor prolonged the retention time of NPs in the colon through bioadhesion (Fig. 4A). The bioadhesion of polyphenol armor comes from the phenolic hydroxyl groups of TA, which is inspired by mussel adhesion. Mussels exhibit strong adhesion to various substrates in seawater via secreted mussel foot proteins, which contain abundant catechol groups. Therefore, siRNA-GBSA(CHI/TA)_5_ NPs have abundant phenolic hydroxyl groups on the polyphenol armor, which interact with colon tissue through electrostatic adsorption, hydrogen bonding, cation-π interaction, and covalent linking (*30*).

To investigate the stability of NPs in the gastrointestinal tract, we first added different NPs into the simulated gastric fluid (SGF) for 2 hours. NPs were then collected and continuously added to the simulated intestinal fluid (SIF) for 4 hours. The ability of NPs to resist the gastrointestinal tract was measured by monitoring the changes in NP morphology, size, and weight. Without armor, siRNA-GBSA NPs immediately turned from turbid to transparent after addition of SGF (fig. S11). Scanning electron microscopy (SEM) showed that siRNA-GBSA NPs were completely degraded in the gastric fluid after 2 hours. siRNA-GBSA(CHI/TA)_2_ NPs exhibited a smaller nanoparticle size than that of their initial state. In contrast, the morphology of siRNA-GBSA(CHI/TA)_5_ NPs remained similar to their initial state after incubation in the SGF for 2 hours. The degradation rates of siRNA-GBSA NPs, siRNA-GBSA(CHI/TA)_2_ NPs, and siRNA-GBSA(CHI/TA)_5_ NPs were 100, 18.6, and 3.7%, respectively (fig. S11).

After continuous incubation in SIF for 4 hours, the total weight loss of the siRNA-GBSA(CHI/TA)_2_ NPs was 39.9% (fig. S11). SEM imaging showed that residual siRNA-GBSA(CHI/TA)_2_ NPs were significantly degraded by SIF and displayed incomplete morphology. In contrast, in the SIF, the total weight loss of siRNA-GBSA(CHI/TA)_5_ NPs was only 9.5% after 4 hours. Moreover, the shape of siRNA-GBSA(CHI/TA)_5_ NPs did not change.

The stability of siRNA-GBSA(CHI/TA)*_n_* NPs (*n* = 2 and 5) was further analyzed in simulated colonic fluid (SCF). The size of siRNA-GBSA(CHI/TA)_5_ NPs was notably reduced when incubated in SCF for 4 hours, but the shape of the NPs remained intact and spherical. The residual siRNA-GBSA(CHI/TA)_2_ NPs displayed various morphologies without specific shapes. After continuous incubation in SCF for 12 hours, the particle size of the siRNA-GBSA(CHI/TA)_5_ NPs decreased but retained an intact morphology. In short, the polyphenol armor allowed siRNA-GBSA NPs to pass through the stomach and the small intestine. Moreover, the thicker armor prevented the rapid degradation of NPs in the small intestine and colonic fluid, prolonging their retention time in the colon.

### Stability of siRNA-GBSA(CHI/TA)_5_ NPs in vivo

The stability of siRNA-GBSA(CHI/TA)_5_ NPs in vivo was explored using normal and mild colitis mice, in which mice were administered a DSS solution (3%, w/v) for 3 days. Fluorescence images of Cy5.5-labeled NPs were acquired using an in vivo imaging system 2, 6, 12, and 24 hours after oral administration. The high fluorescence intensities of mouse abdomens in the DSS + Cy5.5-BSA(CHI/TA)_5_ NP group were maintained for 24 hours (Fig. 4, C and E). In contrast, the fluorescence intensity of the other groups [DSS + Cy5.5, DSS + Cy5.5-BSA NPs, and normal + Cy5.5-BSA(CHI/TA)_5_ NP groups] decreased as time progressed and disappeared after 24 hours. Fluorescence images and quantitative histograms of colon extracts from mice after 24 hours showed that the mean fluorescence intensity of colons in the DSS + Cy5.5-BSA(CHI/TA)_5_ NP group was significantly higher than that of other groups (Fig. 4, D and F). These results prove that polyphenol-armored BSA(CHI/TA)_5_ NPs can smoothly pass through the gastrointestinal tract and ultimately target the inflamed colon.

### Targeted adhesion of inflamed areas in the colon

To confirm that Cy5.5-BSA(CHI/TA)_5_ NPs targeted and adhered to inflamed colon sites, the colons of IBD mice were extracted and incubated with Cy5.5-BSA(CHI/TA)_5_ NPs and Cy5.5-BSACHI NPs for 1 hour. Subsequently, a small-animal fluorescence imaging system was used to characterize the fluorescence intensity of isolated colons after washing three times with phosphate-buffered saline (Fig. 4, G and H). As expected, Cy5.5-BSA(CHI/TA)_5_ NPs showed a more intense fluorescence owing to their negative surface charge and increased bioadhesiveness of phenolic hydroxyl groups compared to Cy5.5-BSA NPs with one CHI layer (Cy5.5-BSACHI NP group), which had a positive surface charge and no phenolic hydroxyl groups.

In summary, the (CHI/TA)_5_ armor can prevent the degradation of siRNA-GBSA(CHI/TA)_5_ NPs in gastric and intestinal fluids, allowing them to reach the colon successfully. siRNA-GBSA(CHI/TA)_5_ NPs have a negative surface charge and good tissue adhesiveness owing to their abundant surface polyphenol structures, which helps them to target and adhere long-term to inflamed colon tissue by hydrogen bonds and electrostatic attractions.

### Excellent therapeutic effect of siRNA-GBSA(CHI/TA)_5_ NPs in DSS-induced colitis mouse model

**Fig. 5.**
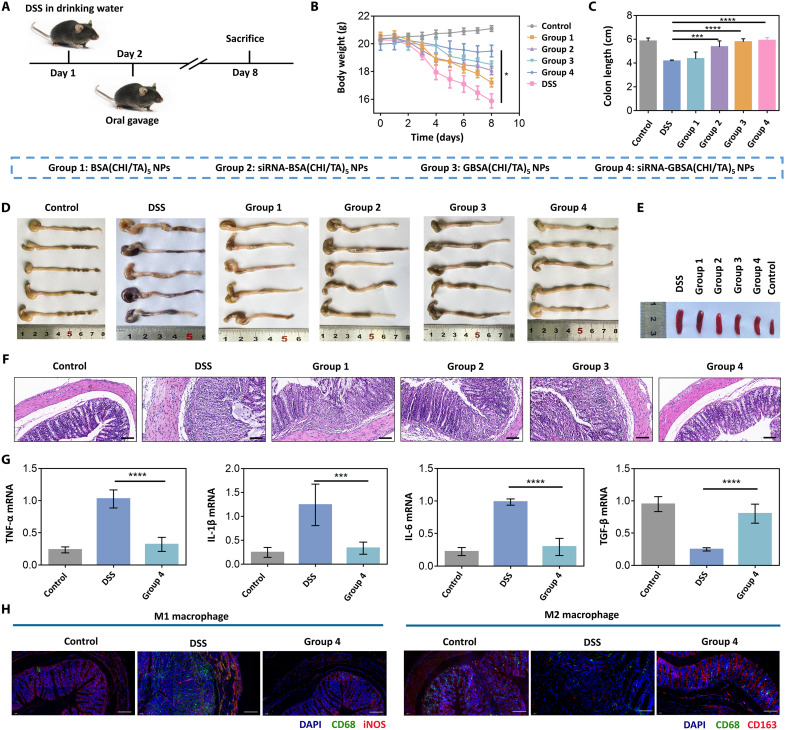
Excellent therapeutic performance of the polyphenol-armored nanomedicine in DSS-induced colitis mouse model. (**A**) Experimental design. Mice were provided sterile water or water containing 3% DSS for 7 days. Treatments were orally administered from the second day. All mice were sacrificed on the eighth day. (**B**) Daily changes in body weight were recorded in detail and analyzed, *n* = 5. (**C** and **D**) Colon lengths were measured and analyzed, *n* = 5. (**E**) Representative macroscopic spleen appearance of each group, *n* = 5. (**F**) Representative hematoxylin and eosin staining images of colon tissue of each group, *n* = 5. Scale bars, 100 μm. (**G**) Colonic mRNA levels of TNF-α, IL-1β, IL-6, and transforming growth factor-β (TGF-β), *n* = 5. (**H**) Immunofluorescence analysis of type 1 macrophages [inducible nitric oxide synthase (iNOS; red), CD68 (green), and 4′,6-diamidino-2-phenylindole (DAPI; blue)] and type 2 macrophage [CD163 (red), CD68 (green), and DAPI (blue)] in colon tissue. Scale bars, 100 μm. (Data are presented as the mean ± SD; *P < 0.05, ***P < 0.001, and ****P < 0.0001).

The DSS-induced colitis mouse model was established by drinking the DSS solution (3%, w/v) for 7 days. NPs gavage was performed on the second day of modeling (Fig. 5A). The results showed that NPs had a potent inflammation-suppressing effect, accompanied by the relief of blood in the stool (fig. S12), weight loss inhibition (Fig. 5B), and recovery of colon length (Fig. 5, C and D) and the spleen (Fig. 5E and fig. S13) compared to untreated colitis mice (DSS group). Histopathological analysis showed that siRNA-GBSA(CHI/TA)_5_ NPs (group 4) elicited better effects against DSS-induced erosion of crypt structures, epithelial damage, and inflammatory cell infiltration than BSA(CHI/TA)_5_ NPs (group 1), nanomedicine without GAGQD [siRNA-BSA(CHI/TA)_5_ NPs; group 2], and GBSA(CHI/TA)_5_ NPs (group 3) (Fig. 5F).

Suppression of inflammation was most efficient with siRNA-GBSA(CHI/TA)_5_ NPs, followed by GBSA(CHI/TA)_5_ NPs, siRNA-BSA(CHI/TA)_5_ NPs, and BSA(CHI/TA)_5_ NPs. The partial anti-inflammatory efficiency of BSA(CHI/TA)_5_ NPs resulted from the ROS-scavenging efficacy of the polyphenol armor. In addition to armor, siRNA-mediated TNF-α gene silencing and the intracellular antioxidant activity of GAGQD promoted the anti-inflammatory efficiency of siRNA-BSA(CHI/TA)_5_ NPs and GBSA(CHI/TA)_5_ NPs, respectively. The siRNA-GBSA(CHI/TA)_5_ NPs exhibited the most potent suppression of inflammation, similar to that observed in normal mice (control group). Compared with siRNA-BSA(CHI/TA)_5_ NPs, GAGQD in siRNA-GBSA(CHI/TA)_5_ NPs scavenge intracellular ROS and protect TNF-α–siRNA from destruction after endocytosis. The intracellular TNF-α–siRNA transfection efficiency was improved by increasing the proportion of intact RNA strands (*36*).

Next, we investigated the in vivo therapeutic mechanism of the siRNA-GBSA(CHI/TA)_5_ NPs. Analysis of myeloperoxidase (MPO) activity in colon tissue showed that MPO activity was significantly reduced by siRNA-GBSA(CHI/TA)_5_ NP treatment compared to that in the DSS group (fig. S14). Moreover, the expression of proinflammatory cytokines (IL-6, IL-1β, and TNF-α) was effectively suppressed, whereas that of the tissue repair–related cytokine transforming growth factor-β (TGF-β) was increased following siRNA-GBSA(CHI/TA)_5_ NP treatment compared with the untreated DSS group (Fig. 5G). Immunofluorescence staining was performed to analyze the macrophage types in the colon after siRNA-GBSA(CHI/TA)_5_ NP treatment (Fig. 5H). The results showed that the number of M1 macrophages was notably decreased and the number of M2 macrophages was substantially increased after siRNA-GBSA(CHI/TA)_5_ NP treatment compared to the DSS group. The siRNA-G BSA(CHI/TA)_5_ NPs were safe in vivo and did not cause systemic toxicity (figs. S15 and S16).

In short, the significant inhibition of TNF-α expression, intracellular antioxidant activity of GAGQD, and extracellular ROS scavenging of the (CHI/TA)_5_ armor synergistically contributed to the excellent inflammation-relieving and tissue repair abilities of siRNA-GBSA(CHI/TA)_5_ NPs. These features allow siRNA-GBSA(CHI/TA)_5_ NPs to effectively treat colitis by reducing MPO activity, inhibiting M1 macrophage polarization, and activating M2 macrophages.

### Modulation of anxiety, depression, and cognition in IBD mice

**Fig. 6.**
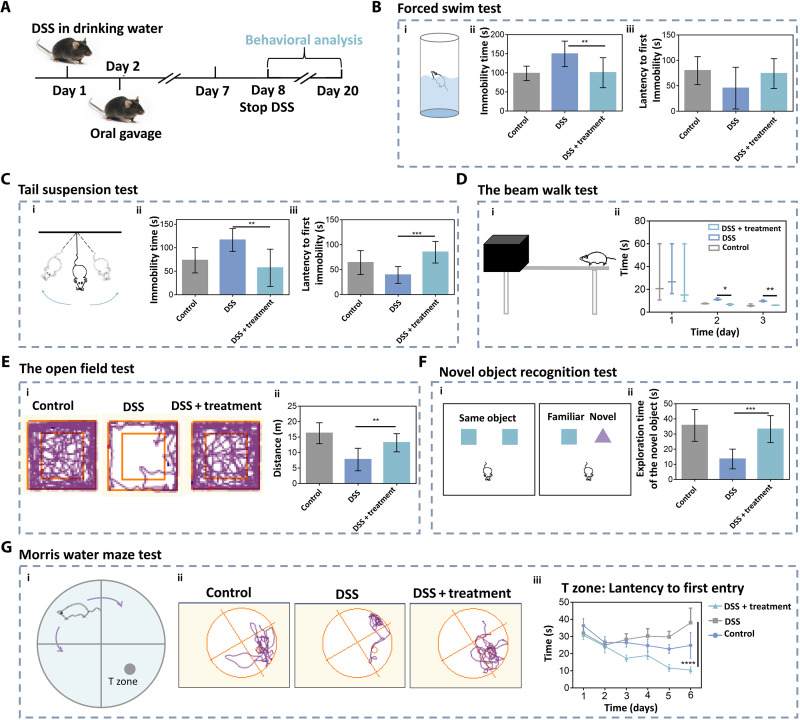
Polyphenol-armored nanomedicine improved learning and cognitive abilities in IBD mice. (**A**) Experimental schedule. siRNA-GBSA(CHI/TA)_5_ NPs were administered on days 2 to 7, and all mice underwent behavioral testing on the indicated days. (**B**) Forced swim test. (i) Schematic of forced swim test, (ii) immobility time, and (iii) latency to first immobility in the forced swim test. (**C**) Tail suspension test. (i) Schematic of tail suspension test, (ii) immobility time, and (iii) latency to first immobility of the tail suspension test. (**D**) The beam walk test. (i) Schematic of the beam walk test; (ii) latency to cross the beam. (**E**) The open field test. (i) Typical escape route map of each group in the open field test; (ii) total distance of each group in the open field test. (**F**) Novel object recognition test. (i) Schematic of novel object recognition test; (ii) exploration time of a new object in the new object recognition test. (**G**) MWM test. (i) Schematic of MWM test, (ii) typical escape route map of each group in MWM test, and (iii) time of entering the T zone for the first time in MWM test. (*n* = 10 mice per group; Data are presented as the mean ± SD; *P < 0.05, **P < 0.01, ***P < 0.001, and ****P < 0.0001).

siRNA-GBSA(CHI/TA)_5_ NPs can relieve IBD-induced psychological symptoms such as depression and anxiety. Here, various behavioral tests, including forced swim (*37*), tail suspension (*38*), beam walk (*39*), open field (*40*), feeding behavior (*41*), novel object recognition (*42*), and Morris water maze (MWM) (*43*), were performed to examine the anxiety, depression, and cognitive behaviors of DSS-induced colitis mice treated with siRNA-GBSA(CHI/TA)_5_ NPs (Fig. 6A). In the forced swim test (Fig. 6B) and tail suspension test (Fig. 6C), the immobility times of mice in the DSS group increased. In contrast, the immobility time of mice in the DSS + siRNA-GBSA(CHI/TA)_5_ NP group decreased, similar to the control group. These results demonstrated that siRNA-GBSA(CHI/TA)_5_ NPs ameliorated depression-like behaviors in IBD mice. The movement and coordination abilities of all the groups were evaluated using the beam-walk test (Fig. 6D). The results showed that the siRNA-GBSA(CHI/TA)_5_ NP-treated group had improved motor coordination. Typical escape routes in the open field test are shown in Fig. 6E. The mice in the DSS group moved along the wall with almost no escape track in the central field, whereas the escape routes in the central field of mice in the DSS + siRNA-GBSA(CHI/TA)_5_ NP group increased, similar to the control group. The total distance moved and the total distance moved in the central field of mice in the DSS + siRNA-GBSA(CHI/TA)_5_ NP group were much higher than that in the DSS group (Fig. 6E and fig. S17). There was no significant difference between DSS + siRNA-GBSA(CHI/TA)_5_ NPs and control groups. In the feeding behavior test, the food intake of IBD mice treated with siRNA-GBSA(CHI/TA)_5_ NP was significantly higher than that of the DSS group and close to that of the control group (fig. S18). These results indicate that siRNA-GBSA(CHI/TA)_5_ NP treatment can improve the feeding behavior of IBD mice.

The novel object recognition test measures learning and memory based on the principle that animals have naturally tend to explore novel objects. DSS-induced colitis mice treated with siRNA-GBSA(CHI/TA)_5_ NPs spent more time exploring novel objects, approaching that of the normal mice (Fig. 6F). However, the mice in the DSS group did not desire to explore new objects. These results suggest that siRNA-GBSA(CHI/TA)_5_ NPs can improve the learning and cognitive function of IBD mice and their motor coordination ability to a certain extent. Typical escape routes in the MWM test are shown in Fig. 6G. The escape routes of mice in the DSS group showed a cluttered escape route with a lower probability in the T zone. In contrast, DSS-induced colitis mice treated with siRNA-GBSA(CHI/TA)_5_ NPs rapidly entered the T zone, and their escape routes circled the cylinder. Moreover, these mice exhibited a significantly lower time to first T zone entry and a significantly longer time spent in the T zone than DSS-induced colitis mice (Fig. 6G and fig. S19). These results suggested that the mice had clear goals and good memory abilities after the treatment with siRNA-GBSA(CHI/TA)_5_ NPs. In short, siRNA-GBSA(CHI/TA)_5_ NPs alleviated colitis and ameliorated IBD-induced mood disorders and cognitive impairments.

### Regulation of bacterial gut microbiota and gut-brain axis in IBD mice

The gut microbiota is a highly varied ecosystem, which consists of a vast majority of microorganisms, such as bacteria, archaea, viruses, and fungi. These gut microbes establish beneficial and symbiotic relationships with the host (*44*). For example, intestinal bacteria exert a protective effect on the host. Normal bacterial flora adhere to, colonize, and propagate in a specific part of the human body to form a membrane barrier, an essential line of defense against the colonization of passing bacteria and plays a critical role in preventing the invasion of foreign pathogenic bacteria (*45*). Fungi are essential component of intestinal microflora. Mucosa-associated fungi can enhance the function of the intestinal epithelium and protect the host from intestinal injury and bacterial infections (*46*). Viruses are crucial regulators of intestinal homeostasis and inflammation. Enteroviruses play an important role in shaping intestinal ecosystems by altering bacterial diversity and promoting horizontal gene transfer (*47*).

**Fig. 7.**
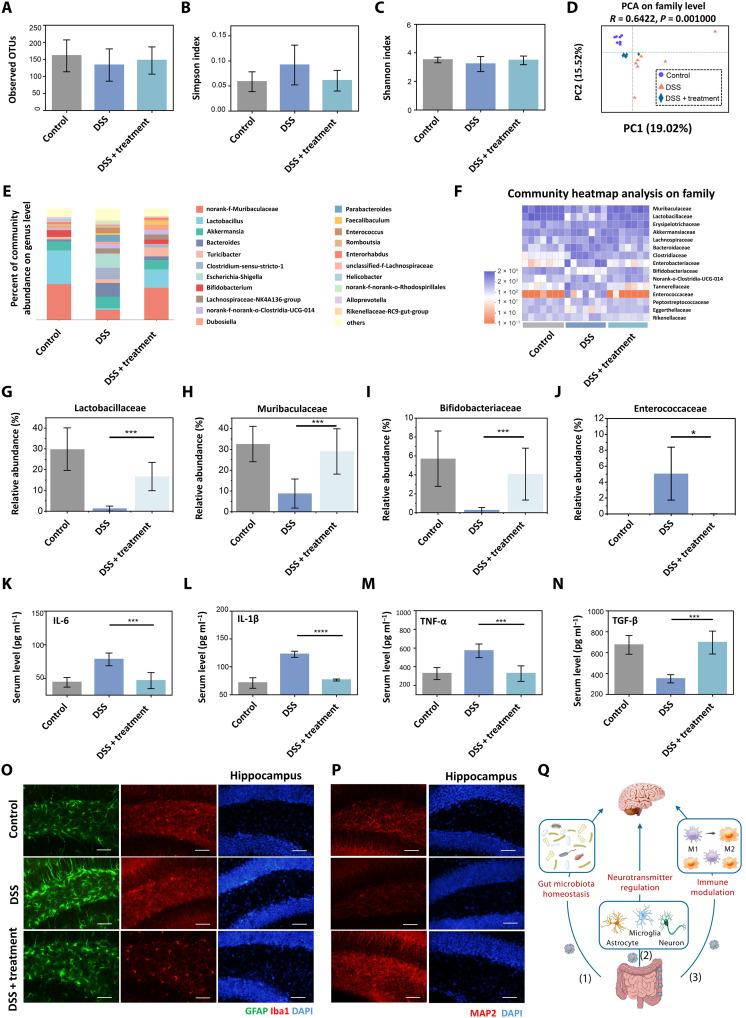
Analysis of gut microbiota–brain interactions regulated by polyphenol-armored nanomedicine. (**A**) Microbial richness [observed operational taxonomic units (OTUs)], (**B**) Simpson index, and (**C**) Shannon index of observed operational taxonomic units showing α-diversity of the microbial community. (**D**) Principal coordinates analysis showing β-diversity of the gut microbiome. Each point represents one mouse and *n* = 7 for each group. The significance of clustering was determined using an analysis of similarities. (**E**) Community histogram at the genus level. Each column represents the mean of each group, *n* = 7. (**F**) Heatmap exhibiting relative abundances of flora at the family level. Each column represents one mouse, *n* = 7. Relative abundance of (**G**) Lactobacillaceae, (**H**) Muribaculaceae, (**I**) Bifidobacteriaceae, and (**J**) Enterococcaceae collected from (F). (**K** to **N**) Serum concentrations of IL-6, IL-1β, TNF-α, and TGF-β, *n* = 7. (**O**) Immunofluorescence staining of GFAP and Iba1 and (**P**) MAP2 in the hippocampal region. Scale bars, 20 μm. (**Q**) Gut microbiota modulates the brain through (1) microbiota homeostasis, (2) neurotransmitters, and (3) immune modulation. (Data are presented as the mean ± SD; *P < 0.05, ***P < 0.001, and ****P < 0.0001, respectively).

Bacteria account for most of these microorganisms. IBD is accompanied by intestinal flora imbalance, affecting intestinal immune homeostasis (*48*). In addition, intestinal microflora can also affect the brain and behavior through the gut-brain axis (*49*). Therefore, to investigate the mechanism by which siRNA-GBSA(CHI/TA)_5_ NPs modulate behavioral changes in the brain, an in-depth analysis of fecal samples from mice with DSS-induced colitis was performed using advanced 16*S* ribosomal RNA (rRNA) sequencing technology. Flora richness and α diversity were not significantly different between the control, DSS, and DSS + siRNA-GBSA(CHI/TA)_5_ NP groups, probably because of the short modeling period (Fig. 7, A to C). However, the principal coordinate analysis indicated that siRNA-GBSA(CHI/TA)_5_ NPs substantially altered the composition of the microflora in DSS-induced colitis mice (Fig. 7D), which was similar to the control group. The general enterobacterial composition at the genus (Fig. 7E) and family levels (Fig. 7F) for each sample are shown by bar graphs and heatmaps, respectively. Oral administration of siRNA-GBSA(CHI/TA)_5_ NPs helped maintain a similar bacterial microbiota composition at the genus level compared to the control group. The abundances of norank-f-Muribaculaceae and Lactobacillus were notably increased compared with those in the DSS group, similar to the trend of bacterial microbiota in cured IBD patients reported in the literature (*50*).

Next, several particular bacteria were quantitatively analyzed at the family level (Fig. 7, G to J). Treatment with siRNA-GBSA(CHI/TA)_5_ NPs significantly retained the relative abundance of Lactobacillaceae (known for their beneficial roles in both IBD animal models and patients) (*51*), Muribaculaceae (a major mucin monosaccharide forager that acts as an ecological gatekeeper in healthy guts) (*52*), and Bifidobacteriaceae (a common intestinal probiotic species that regulates intestinal flora) (*53*). In addition, Enterococcaceae, a typical class of opportunistic pathogens in the intestines of humans and other animals, were markedly decreased. These results suggest that siRNA-GBSA(CHI/TA)_5_ NPs positively modulate the gut microbiota to enhance the local immune microenvironment.

Intestinal microorganisms and activated immune cells in the intestinal lumen produce numerous inflammatory factors that enter blood circulation through the damaged intestinal mucosal barrier and stimulate other immune cells to produce inflammatory cytokines (*54*). Peripherally produced inflammatory factors can increase the permeability of the blood-brain barrier, which can directly affect the brain (*55*). Therefore, the detection of systemic inflammatory factors is necessary to understand the changes in brain inflammation in IBD mice before and after siRNA-GBSA(CHI/TA)_5_ NP treatment. In this study, we collected blood from mice with orbital bleeding and performed enzyme-linked immunosorbent assay (ELISA). The results showed that proinflammatory factors in the blood, including IL-6, IL-1β, and TNF-α, increased sharply after 7 days of DSS administration. After treatment with siRNA-GBSA(CHI/TA)_5_ NPs, the levels of proinflammatory factors in the blood decreased, approaching normal levels (Fig. 7, K to N). These results indicated that siRNA-GBSA(CHI/TA)_5_ NPs relieved intestinal inflammation, regulated intestinal flora, and inhibited systemic inflammation.

Next, we explored the effects of siRNA-GBSA(CHI/TA)_5_ NPs on brain inflammation and neurons in IBD mice. First, immunofluorescence staining was performed on the hippocampus (Fig. 7O) using glial fibrillary acidic protein (GFAP) to label astrocytes. The results showed that the intensity of GFAP labeling was higher in the DSS group, and astrocytes were substantially activated, indicating that DSS treatment caused severe brain inflammation. The GFAP labeling intensity in the DSS + siRNA-GBSA(CHI/TA)_5_ NP group was notably decreased, and there was no significant difference compared to the control group.

Ionized calcium-binding adapter 1 (Iba1) is a microglia-specific calcium-binding protein used to label microglia. The microglia in the DSS group displayed strong fluorescence, enlarged cell bodies, and thickened axons. In the DSS+ siRNA-GBSA(CHI/TA)_5_ NP group, microglial processes were slender and displayed normal inclusion bodies (Fig. 7O).

Immunofluorescence staining of neurons in the hippocampus (Fig. 7P) and the cerebral cortex (fig. S20) was performed by labeling mature neurons with microtubule-associated protein 2 (MAP2). The results showed notably weak MAP2 fluorescence in the hippocampus and cerebral cortex of the DSS group, and nerve cells were disordered and fractured, with a marked loss of neurons. After treatment with siRNA-GBSA(CHI/TA)_5_ NPs, the fluorescence intensities of hippocampus and cerebral cortex neurons were substantially increased, similar to those in the control group. In addition, the connections between neurons were more apparent than in the DSS group, and there were no breakages. These results indicate that treatment with siRNA-GBSA(CHI/TA)_5_ NPs notably reduced brain inflammation and repaired lost and damaged neurons.

We next explored the effects of siRNA-GBSA(CHI/TA)_5_ NPs on the regulation of gut microbiota and gut-brain axis in IBD mice. The 16*S* rRNA sequencing results showed that Lactobacillus is one of the most beneficial bacteria in the intestine (fig. S21). The relative abundance of Lactobacillus at the genus level in the DSS group was low. However, it increased significantly after siRNA-GBSA(CHI/TA)_5_ NP treatment, close to that in the control group. As a beneficial bacterium, Lactobacillus maintain intestinal homeostasis and have a close relationship with anxiety and depression (*56*). We next explored the effects of siRNA-GBSA(CHI/TA)_5_ NPs on the central nervous system (CNS) of IBD mice. The mRNA expression levels of γ-aminobutyric acid (GABA), the primary inhibitory neurotransmitter in the CNS, were determined using real-time polymerase chain reaction (RT-PCR). Although no significant expressions of GABA_Aα1_ in the hippocampus and GABA_B1_ in the hippocampus and cerebral cortex were detected, the levels of GABA_Aα1_ in the prefrontal cortex and cerebral cortex and GABA_B1_ in the prefrontal cortex in the DSS group were the highest. Similar to the control group, a significantly lower level was found after siRNA-GBSA(CHI/TA)_5_ NP treatment. The above results show that brain function can be improved by siRNA-GBSA(CHI/TA)_5_ NP treatment. In summary, siRNA-GBSA(CHI/TA)_5_ NPs increased the abundance of Lactobacillus and inhibited the expression of GABA receptors through the gut-brain axis. This means that bacteria play an essential role in the two-way communication of the gut-brain axis.

In short, our results show a bidirectional interaction between the bacterial gut microbiota and the brain. The mechanism by which siRNA-GBSA(CHI/TA)_5_ NPs regulate bacterial gut microbiota–brain interactions can be attributed to three pathways (Fig. 7Q): (1) TA and its microbial metabolites increase the diversity of bacterial gut microbiota and abundance of beneficial flora; (2) NPs modulate neurotransmitters to inhibit activated astrocytes and microglia, restore damaged and lost neurons in the brain; and (3) NPs regulate intestinal immune responses to reduce systemic inflammation.

## DISCUSSION

We developed polyphenol-armored siRNA-GBSA(CHI/TA)_5_ NPs capable of treating colitis, maintaining bacterial gut microbiota homeostasis, and modulating mood and cognitive dysfunctions. Under the protection of (CHI/TA)_5_, siRNA-GBSA(CHI/TA)_5_ NPs successfully passed through the gastrointestinal tract and were enriched long-term in inflamed colon sites, owing to the negative surface charge and tissue adhesion of the armor. The (CHI/TA)_5_ armor was slowly degraded at the colonic site, allowing TA to be catabolized by microbiota into more active phenolic metabolites. Meanwhile, siRNA-GBSA(CHI/TA)_5_ NPs achieved efficient endocytosis, owing to the high cellular affinity of TA. After endocytosis, GAGQD and TNF-α–siRNA were released under the action of intracellular enzymes. GAGQD ensured the integrity of the TNF-α–siRNA chain by removing intracellular ROS, allowing TNF-α–siRNA to silence TNF-α expression effectively. These features of NPs synergistically suppressed intestinal inflammation, restored intestinal flora and immune homeostasis, and improved anxiety, depression, and cognitive behavior. Overall, this polyphenol-armored strategy offers a universal, powerful platform for the design of oral nanomedicines for inflammatory diseases.

## MATERIALS AND METHODS

### Materials

Citric acid monohydrate (CA) (C_6_H_8_O_7_·H_2_O, 99.5%), GA, ethylenediamine [NH_2_(CH_2_)_2_NH_2_], BSA, TA, CHI, glutaraldehyde, and ethanol were purchased from Sigma-Aldrich (St. Louis, MO, USA). TNF-α siRNA (sense: 5′-CACAACCAACUAGUGGUGCUU-3′; antisense: 5′-AAGCACCACUAGUUGGUUGUG-3′) and Cy5.5-siRNA (sense: 5′Cy5.5-UUCUCCGAACGUGUCACGUTT-3′; antisense: 5′-ACGUGACACGUUCGGAGAATT-3′) were synthesized by Gene Pharma Co. Ltd. (Shanghai, China). All purchased reagents and siRNAs were used as received, unless otherwise stated.

### Synthesis of GAGQD

GAGQDs were prepared according to a previous report (*57*) with some modifications. CA (2 g) and GA (2 g) were dissolved in 60 ml of deionized (DI) water, after which 100 μl of ethylenediamine was added to the mixture and stirred for 10 min. The mixture was immediately transferred to a 100-ml stainless steel reactor with a Teflon liner and subjected to a hydrothermal reaction at 180°C for 6 hours. After cooling to 37°C, the black-yellow product obtained was centrifuged at 10,000 rpm for 20 min to remove large particles, and the supernatant was collected. The supernatant was transferred to a dialysis kit [molecular weight cutoff (MWCO) 1000] and dialyzed against 3 liters of DI water for 48 hours. During the dialysis process, DI water was replaced eight times. Last, GAGQD powder was obtained after rotary evaporation and freeze-drying. The synthesis of GQD was the same as that of GAGQD, except that GA was not used.

### Synthesis of TNF-α–siRNA and GAGQD-encapsulated bovine serum albumin nanoparticles (siRNA-GBSA NPs)

siRNA-GBSA NPs were prepared according to previous reports, with some modifications (*58*). BSA (20 mg), GAGQD (0.5 mg), and TNF-α–siRNA (62 μg) were dissolved in 1 ml of NaCl (10 mM) solution and stirred at 25°C for 1 hour. The pH of the mixed solution was adjusted to 8 by adding 1 M NaOH solution, after which 4 ml of ethanol was slowly added, and the mixture was stirred at room temperature for 3 hours. Thereafter, glutaraldehyde solution (8%, 16 μl) was added dropwise to the mixed solution, followed by stirring at room temperature for 24 hours. The final product was centrifuged (12,000 rpm, 30 min) and washed thrice with a 50% water/ethanol mixture to obtain purified siRNA-GBSA NPs.

### Preparation of (CHI/TA)*_n_* armor on siRNA-GBSA NPs [siRNA-GBSA(CHI/TA)*_n_* NPs]

siRNA-GBSA(CHI/TA)*_n_* NPs were prepared by a LbL assembly approach using CHI and TA. The specific steps are as follows.

Ten milliliters of siRNA-GBSA NP solution (1 mg ml^−1^) was ultrasonicated for 30 min to disperse the solution evenly. The CHI solution (10 ml, 1 mg ml^−1^) was quickly added to the siRNA-GBSA NP solution and stirred for 30 min. The mixed solution was then centrifuged at 12,000 rpm for 10 min and washed thrice with DI water to obtain CHI-coated siRNA-GBSA NPs (siRNA-GBSACHI NPs).

The obtained siRNA-GBSACHI NPs were dissolved in 10 ml of DI water and sonicated for 30 min. TA solution (10 ml, 1 mg ml^−1^) was quickly added to the siRNA-GBSACHI NP solution and stirred for 30 min. The mixed solution was centrifuged at 12,000 rpm for 10 min and washed thrice with DI water to obtain TA-coated siRNA-GBSACHI NPs [siRNA-GBSA(CHI/TA)_1_]. siRNA-GBSA(CHI/TA)*_n_* NPs were obtained by alternating the self-assembly of CHI and TA in *n* cycles.

### Characterization

TEM images of GAGQD were obtained using a JEM-2100F field-emission transmission electron microscope at an acceleration voltage of 200 kV. The morphologies of the different NPs were characterized by SEM (Zeiss Supra 55). The chemical bonds of GAGQD were analyzed using an x-ray photoelectron spectroscope (Thermo Fisher Scientific, ESCALAB250) with Al-Kα x-ray radiation as the excitation source (1486.8 eV, 500 nm). UV-vis spectroscopy was performed using a Lambda 950 UV-vis spectrophotometer. FTIR was performed using a Nicolet 5700 FTIR spectrometer. The luminescence spectra were acquired using a fluorescence spectrometer (FL-4600, Hitachi). All the measurements were performed at room temperature. The particle size and zeta potential of the NPs were measured using a highly sensitive zeta potential and particle size analyzer (Nano ZS).

### Superoxide dismutase activity of NPs

Superoxide dismutase (SOD) activity of the NPs was assessed using a SOD activity detection kit (Solarbio) according to the manufacturer’s instructions. The superoxide anion (•O_2_^−^) generated by the xanthine-xanthine oxidase system, which reduces nitroblue tetrazolium to blue formazan, absorbs light at 560 nm. SOD scavenges •O_2_^−^, thereby inhibiting formazan formation.

GQD, GAGQD, GBSA NPs, GBSA(CHI/TA)_5_ NPs, and the reagents provided in the kit were added to the 96-well plate according to the manufacturer’s instructions, with a total volume of 200 μl per well. After 30 min in a water bath at 37°C, the absorbance was measured at 560 nm using a microplate reader. The elimination rate of •O_2_^−^ was calculated by measuring the decrease in color development. The final concentrations of GQD, GAGQD, GBSA NPs, and GBSA(CHI/TA)_5_ NPs were 100 μg ml^−1^.

### Hydroxyl radical scavenging activity

The hydroxyl radical (•OH) scavenging activities of the GQD, GAGQD, GBSA NPs, and GBSA(CHI/TA)_5_ NPs were evaluated by electron paramagnetic resonance (EPR) analysis using a Bruker EMXPlus spectrometer (Bruker EMXPlus-10/12). •OH was trapped by 5,5-dimethyl-1-pyrroline *N*-oxide (DMPO) in an aqueous solution to form a spin adduct, DMPO/OH. •OH was generated using the Fenton reaction, and the electron spin resonance signals were obtained by mixing 25 mM BMPO, 1.0 mM FeSO_4_, and 1.0 mM H_2_O_2_ in water. In the •OH scavenging activity experiments, EPR spectra of DMPO/OH were recorded after 0- and 300-s incubation of the newly generated •OH, DMPO, and samples. The concentrations of GQD, GAGQD, GBSA NPs, and GBSA(CHI/TA)_5_ NPs were 20 μg ml^−1^. The typical EPR spectrum of DMPO/OH showed four lines with a relative intensity of 1:2:2:1; therefore, the intensity of the second line was used to calculate the elimination rate of the sample.

### Stability of siRNA-GBSA(CHI/TA)*_n_* NPs in SGF, SIF, and SCF

The stability of NPs in the simulated gastrointestinal fluid was evaluated as previously described, with modifications (*59*). Briefly, 1 ml of freshly prepared siRNA-GBSA NPs, siRNA-GBSA(CHI/TA)_2_ NPs, and siRNA-GBSA(CHI/TA)_5_ NPs (10 mg ml^−1^) was diluted (1:1, v/v) with SGF [(pH 2) containing pepsin (10 mg ml^−1^)] and incubated for 2 hours at 37°C. The NPs in the SGF were centrifuged (10,000 rpm for 10 min). The precipitate was dispersed in SIF (pH 6.8) and incubated at 37°C for 4 hours. The NP/SIF solution was then centrifuged (10,000 rpm, 10 min), and the precipitate was dispersed in 1 ml of SCF (pH 7.8) and incubated at 37°C for a specific time.

The NP/simulated solution was centrifuged (10,000 rpm, 10 min) and washed with DI water two to three times, and the remaining NPs were weighed after drying. The degradation rates of NPs at different stages were calculated as follows: degradation rate (%) = (initial weight − remaining weight)/initial weight.

### Stability of NPs in gastrointestinal tract

To avoid autofluorescence from different tissues, the NPs were labeled with the near-infrared fluorescent dye Cy5.5 to evaluate the stability of the NPs after oral administration. After 3 days of feeding with 3% DSS (Millipore), 200 μl of Cy5.5 (1 mg ml^−1^), Cy5.5-BSA NPs (5 mg ml^−1^), and Cy5.5-BSA(CHI/TA)_5_ NPs (5 mg ml^−1^) was orally administered to the mice after fasting for 48 hours. At different periods (2, 6, 12, and 24 hours), abdominal fluorescence in the mice was recorded using an in vivo imaging system (IVIS) small animal fluorescence imaging system (Lumina III). The mice were sacrificed 24 hours after NP gavage, and the removed colon was imaged using IVIS small-animal fluorescence imaging to observe the retention of NPs in the colon.

### Animal and DSS-induced mouse models

C57BL/6 mice (female, 8 weeks old) were obtained from Chengdu Dashuo Biotechnology Co. Ltd. All animal experiments were performed according to protocols approved by the local ethics committee and the laboratory animal administration rules of China (IACUC-DWZX-2021-759).

All mice (*n* = 102) were housed in a specific pathogen-free environment. Mice were randomly divided into six groups. Acute colitis was induced by DSS administration for 7 days. The control group received sterile water without DSS. The remaining five groups were treated with DSS (DSS group), BSA(CHI/TA)_5_ NPs (group 1), siRNA-BSA(CHI/TA)_5_ NPs (group 2), GBSA(CHI/TA)_5_ NPs (group 3), and siRNA-GBSA(CHI/TA)_5_ NPs (group 4) and received DSS solution (3%, w/v) freshly prepared every 2 days. NP gavage (1 mg ml^−1^, 200 μl) was performed on the second day after colitis induction. During this period, mice were observed daily for fecal consistency, blood in the stool, and body weight. On the eighth day, NP gavage and DSS feeding were discontinued, and DSS water was replaced with sterile water without DSS. Ten mice in each group were reserved for behavioral analysis, and the remaining seven mice in each group were euthanized. Blood, brain, fecal (colon site), colon, and spleen samples were collected for further analysis.

### Quantitative PCR

Cells were collected in TRIzol (Sigma-Aldrich), and total RNA analysis was performed for samples prepared by chloroform extraction and isopropanol precipitation, according to the manufacturer’s recommendations (Invitrogen). cDNA was used as a template for quantitative PCR with SYBR Green (Toyobo) to determine the expression of specific genes. RT-PCR using SYBR Green PCR mix was conducted using an FTC-3000 system. The primer sequences used were as follows: IL-1β (forward, GTGGCTGTGGAGAAGCTGTGG; reverse, CGGAGCCTGTAGTGCAGTTGTC), IL-6 (forward, 5′-ACTTCCATCCAGTTGCCTTCTTGG-3′; reverse, 5′-TTAAGCCTCCGACTTGTGAAGTGG-3′), TNF-α (forward, 5′-GATGGGTTGTACCTTGTCTACT-3′; reverse, 5′-CTTTCTCCTGGTATGAGATAGC-3′), TGF-β (forward, 5′-CCAGATCCTGTCCAAACTAAGG-3′; reverse, 5′-CTCTTTAGCATAGTAGTCCGCT-3′), GABA_Aα1_ (forward, 5′-ACTGCTGGACGGTTATGACAATCG-3′; reverse, 5′-GGTCTGAAACTGGTCCGAAACTGG-3′), and GABA_b1_ (forward, 5′-CCATCCGCCACACTCCACAATC-3′; reverse, 5′-TTCACTCGCTCCTCCAGGTCATC-3′).

### Quantification of inflammatory cytokines in blood

After various treatments, serum samples were isolated from mice and diluted for analysis. The serum levels of TNF-α, IL-1β, IL-6, and TGF-β were measured using ELISA (eBioscience).

### Statistical methods

Data are presented as the mean ± SD. Significant differences were analyzed using one-way analysis of variance (ANOVA) followed by Tukey’s post hoc test. Data are presented as mean ± SEM (*n* ≥ 3); *P < 0.05, **P < 0.01, ***P < 0.001, and ****P < 0.0001.

## Notes

View/request a protocol for this paper from *Bio-protocol*.
