# Supplementary material for: Oral polyphenol-armored nanomedicine for targeted modulation of gut microbiota–brain interactions in colitis
Source: Sci Adv. 2023 May 26;9(21):eadf3887. doi: 10.1126/sciadv.adf3887 (PMC10219598; doi:10.1126/sciadv.adf3887)
Supplement: Supplementary file 1 — Supplementary Text Fig. S1 to S21 [file sciadv.adf3887_sm.pdf]

Science Advances
AAAS

Supplementary Materials for

Oral polyphenol-armored nanomedicine for targeted modulation of gut microbiota–brain interactions in colitis

Huan He *et al.*

Corresponding author: Chao Wang, cwang@suda.edu.cn; Xiaoxia Jiang, smilovjiang@163.com; Xiong Lu, luxiong_2004@163.com; Chaoming Xie, xie@swjtu.edu.cn

Sci. Adv. **9**, eadf3887 (2023)
DOI: 10.1126/sciadv.adf3887

This PDF file includes:

Supplementary Text
Fig. S1 to S21

Supplementary Text

Preparation of Cy5.5-siRNA-GBSA NPs for encapsulation efficiency test

BSA (20 mg), GAGQD (0.5 mg), and Cy5.5-siRNA (62 µg) were dissolved in 1 mL of NaCl (10 mM) solution and stirred at room temperature for 1 h. The pH of the mixed solution was adjusted to 8 using 1 M NaOH solution, followed by slow addition of 4 mL of ethanol, after which the mixture was stirred at room temperature for 24 h. The final product was centrifuged (12000 rpm, 10 min) and washed thrice with a 50 % water/ethanol mixture to obtain purified Cy5.5-siRNA-GBSA NPs.

Preparation of Cy5.5-BSA NPs, Cy5.5-BSACHI NPs, Cy5.5-BSA(CHI/TA)$_2$ NPs, and Cy5.5-BSA(CHI/TA)$_5$ NPs

BSA (100 mg) was dissolved in 5 mL of NaCl (10 mM) solution and stirred at room temperature for 1 h. The pH of the mixed solution was adjusted to 8 by adding 1 M NaOH solution, after which 20 mL of ethanol with 2.5 mg Cyanine5.5 NHS ester (Cy5.5) (A3009, APExBIO) was slowly added, and the mixture was stirred at room temperature for 3 h. Glutaraldehyde solution (8 %, 80 µL) was added dropwise to the above mixed solution, followed by stirring at room temperature for 24 h. The final product was centrifuged (12000 rpm, 30 min) and washed thrice with a 50 % water/ethanol mixture to obtain purified Cy5.5-BSA NPs.

Cy5.5-BSA(CHI/TA)$_2$ NPs and Cy5.5-BSA(CHI/TA)$_5$ NPs were prepared from CHI and TA via an LbL assembly approach using two and five cycles. Cy5.5-BSACHI NPs were obtained by electrostatically assembling a CHI layer on the surface of the Cy5.5-BSA NPs.

Encapsulation efficiency of siRNA and GAGQD in BSA NPs

First, Cy5.5-siRNA-GBSA NPs were dissolved in DI water at 5 mg mL^{-1} and placed at 37 °C for 4 h until the solution became transparent. The absorption of the solution at 675 nm (Cy5.5-siRNA) and 350 nm (GAGQD) was measured using an ultramicro nucleic acid analyzer (Nano-400A) and a UV–vis spectrophotometer. The encapsulation efficiency was calculated according to the concentration-based standard curves of Cy5.5-siRNA and GAGQD.

Cell experiment

RAW264.7 cells were cultured in high-glucose Dulbecco's modified Eagle's medium (high-glucose DMEM; HyClone, USA) containing 10 % fetal bovine serum (HyClone) and 1 % penicillin-streptomycin solution (HyClone) in a 37 °C CO_2 incubator.

Cytotoxicity assay

The cytotoxicity of the siRNA-GBSA(CHI/TA)$_5$ NPs to RAW264.7 cells was assessed using the 3-(4,5-dimethylthiazol-2-yl)-2,5-diphenyltetrazolium bromide (MTT) assay. First, 500 µL of RAW264.7 cells (1 × 10^5 cells mL^{-1} in high-glucose DMEM medium) was added to each well of a 48-well plate and incubated in a 5 % CO_2 incubator at 37 °C until fully attached. The medium was then removed, and 500 µL of high-glucose DMEM with different concentrations of siRNA-GBSA(CHI/TA)$_5$ NPs (0, 100, 200, 300, 500, 600, 800, and 1000 µg mL^{-1}) was added to the corresponding wells. After 24 h of incubation, 100 µL of 5 mg mL^{-1} MTT solution was added to each well and incubated at 37 °C for 4 h. After removing the medium, 200 µL of dimethyl sulfoxide was added to each well and incubated for 20 min. Finally, the absorbance of the medium was measured at 490 nm using an ELISA microplate reader. The cell viability is expressed as a

percentage of the absorbance relative to that of the control (no NPs). Here, siRNA-GBSA(CHI/TA)$_5$ NPs were sterilized by immersion in 75 % ethanol for 24 h.

Oxidative stress assay

RAW264.7 cells were seeded in 24-well plates with cell slides at a density of 1.5×10^4 cells per well. After cell adherence, the medium was removed and the high-glucose DMEM medium containing lipopolysaccharide (LPS) (L4391, Sigma, USA) (100 ng mL^{-1}) and NPs (200 μg mL^{-1}) was added and incubated with the cells for 24 h. BSA NPs, GBSA NPs, siRNA-GBSA NPs, and siRNA-GBSA(CHI/TA)$_5$ NPs were used for the analysis. Thereafter, the cells were washed with PBS and stained with the ROS-sensitive probe 2',7'-dichlorodihydrofluorescein diacetate (DCFH-DA, 500 μL, 10 μM). Finally, the characteristic fluorescence of ROS in the cells was observed using a confocal laser scanning microscope (CLSM, LSM880, Zeiss, Germany) after washing the cells three times with PBS.

Flow cytometry was used to quantify the intracellular ROS levels. After DCFH-DA staining, the cells were washed three times with PBS, scraped off the well, and dispersed in 1 mL PBS for detection on a flow cytometer (Cytoflex, Beckman, USA).

Endocytosis experiments

RAW264.7 cells (1 mL; 1×10^5 cells mL^{-1}) were seeded in confocal dishes and incubated for 12 h. The cell culture medium was then changed to 1 mL of fresh high-glucose DMEM medium containing 200 μg mL^{-1} of Cy5.5-siRNA, Cy5.5-siRNA-GBSA NPs, Cy5.5-siRNA-GBSACHI NPs, Cy5.5-siRNA-GBSA(CHI/TA)$_5$ NPs, and Cy5.5-siRNA-GBSA(CHI/TA)$_1$ NPs, respectively,

and incubated with the cells for 4 or 12 h. The culture medium was removed, and 4,6-diamidino-2-phenylindole (DAPI; ab1041139, Abcam) was added to each well for nuclear labeling. The cells were observed using CLSM after repeated washing with phosphate-buffered saline (PBS). The cells were gently scraped and collected in 1 mL PBS solution for quantitative flow analysis.

Anti-inflammatory activity *in vitro*

RAW264.7 cells were seeded into 12-well culture plates (10^7 cells per well). The medium was then changed to high-glucose DMEM containing LPS (500 ng mL^{-1}) and 200 μg mL^{-1} NPs (BSA $(CHI/TA)_1$ NPs, siRNA-BSA$(CHI/TA)_1$ NPs, GBSA$(CHI/TA)_1$ NPs, or siRNA-GBSA$(CHI/TA)_1$ NPs). After 24 h of treatment, the supernatant was collected, and inflammatory factors, including tumor necrosis factor-α (TNF-α), interleukin-6 (IL-6), arginase-1 (Arg-1), and interleukin-10 (IL-10), were tested by ELISA according to the manufacturer's protocol (eBioscience).

Determination of myeloperoxidase activity

Colon tissue was homogenized in ice-cold potassium phosphate buffer (pH 6.0) containing 0.5 % cetyltrimethylammonium bromide. The homogenate was sonicated, freeze-thawed three times, centrifuged at 3000 rpm for 15 min at 4 °C, and kept at −80 °C until analysis. The myeloperoxidase (MPO) activity was determined using an MPO assay kit (ab111749; Abcam). Briefly, 10 µL of the supernatant was transferred to PBS (pH = 6.0) containing 3,3'-dimethoxybenzidine (0.17 mg mL^{-1}) and hydrogen peroxide (0.0005 %). The MPO activity was determined by measuring the hydrogen peroxide-dependent oxidation of 3,3'-dimethoxybenzidine, expressed in units per gram of total protein (U/g). The total protein content of the corresponding samples was analyzed using a bicinchoninic acid (BCA) protein assay kit (Sigma-Aldrich).

Immunochemistry

Colon tissue samples were fixed with 4 % paraformaldehyde (Biosharp). The tissue was embedded in paraffin and sectioned at a thickness of 4 μm after dehydration in ethanol. Histopathological features were detected using hematoxylin and eosin (Solarbio) staining. For immunohistochemical staining, tissue sections were deparaffinized, rehydrated, and rinsed, followed by antigen retrieval (heat-induced epitope retrieval method) and blocking (goat serum (Solarbio)). The tissue sections were incubated overnight with primary antibodies, followed by incubation with biotinylated secondary antibodies (for immunohistochemical staining). ADAB horseradish peroxidase chromogenic kit (Dako, Agilent Technologies, USA) was used for the chromogenic reaction in the immunohistochemical staining. Finally, the tissue sections were observed under a fluorescence microscope (Leica fluorescence optical microscope DM4000). The following primary antibodies were used: CD68 (1:500; Abcam), CD163 (1:5000; Abcam)), and iNOS (1:1000; Proteintech).

Immunofluorescence

The mice were anesthetized with 2,2,2-tribromoethanol (350 mg kg^{-1}, Sigma-Aldrich, T48402), perfused transcardially with 0.9 % saline, and their brains were removed and fixed in phosphate-buffered 4 % paraformaldehyde for 48 h, followed by cryoprotection with 30 % sucrose. The mouse brain slices (30 μm) were washed three times with PBS. The samples were then permeabilized and blocked in PBS containing 0.3 % Triton X-100 and 10 % normal goat serum for 1 h at room temperature. Sections were incubated with the primary antibody in blocking buffer overnight at 4 °C. After washing, the secondary antibodies were added to the blocking buffer and incubated for 1 h at room temperature. The samples were then washed and counter-stained with

DAPI. Images were acquired using a fluorescence microscope (Nikon AZ-100 Multifunction Microscope). The primary antibodies used for immunostaining included GFAP (EMD Millipore, 3,380,386, Mouse), IBA1 (rabbit, 1:300; Abcam, ab178847), and MAP2 (Rabbit, 1:500; Abcam, ab32454). Donkey anti-mouse/rabbit 488/594 secondary antibodies (1:1000) and DAPI-containing mounting medium were purchased from Invitrogen.

Behavioral analysis of mice

Forced swim test (FST)

The FST was used as a behavioral endorsement test to assess desperate behavior, in which an increase in the immobility time represents depression-like behavior. Mice were placed individually in an open glass cylinder (diameter: 10 cm, height: 25 cm) filled with 19 cm of water (23±1 °C). The mice were allowed to swim for 6 min, and the immobility time was recorded for the last 4 min. The immobility behavior was measured when the animal floated motionless in the water and performed only the activities necessary to keep its head above the water.

Tail suspension test (TST)

The tail suspension test is also used to evaluate the behavior that related to depression in mice. In tail suspension experiment, the immobility time of tail suspension mice was used as an indicator to detect the despair behavior of animals. In the TST, the mice were suspended on the edge of a rod 50 cm above a tabletop using adhesive Scotch tape located approximately 1 cm from the tip of the tail. Tail climbing was prohibited by passing the mice tail through a small plastic cylinder before hanging. The duration of the immobility time was recorded manually over 6 min. mice were considered immobile only when they hung passively and were completely immobile.

Beam walk test

The balance beam test is an experiment to study the balance ability of animals and detect their motor coordination. The time is recorded when the mice reach the small black box through the balance beam. If it fails to reach the small magazine, the time is recorded as 60 s. To examine inflammatory bowel disease (IBD)-associated complex motor movements and coordination, a beam-walking test was performed, as previously described, with some modifications. The test was performed 1–7 days post-IBD. Briefly, the beam was a wooden bar (length: 1200 mm, width: 21 mm) placed above the ground. A black box was used for animal acclimatization. The mice were allowed to walk on the beam to the box and remain in the box for 60 s. Thereafter, they were placed on the beam at a starting distance of 35 cm from the box. The mice were allowed to walk to the box and stay there for 60 s. This step was repeated. The next day, the mice were placed in the box for 60 s and then allowed to go to the box, with the starting point initially at 35 cm; the distance from the box was gradually increased to 100 cm. The beam crossing experiment was repeated three times, and the mice were allowed to rest in the box for 1 min. The mean score was calculated from three runs per day. The time to cross the beam of the test was counted offline by an observer blinded to the animal treatments.

Open field test

Open field experiment is a method to evaluate the autonomous movement behavior. During the experiment, the mice were put into a specific position in the open field, and the camera system was used to monitor the activities of the mice in the open field. The mice with lower anxiety tended to stay in the center of the open field for more time. Therefore, the anxiety state of mice was evaluated by recording the time of mice's central exploration in this article. In this task, anxiety is reflected

by the amount of time the rodents spend at the edges of the box, avoiding the center of the open field. The total distance travelled, entries within each zone, and the time spent and distance travelled within each zone were recorded over the course of 20 min. Data were recorded using a video tracking system (SMART, Panlab Harvard Apparatus, Bioscience Company, Holliston, MA, USA).

New object recognition test

New object recognition test is a learning and memory test method based on the principle that animals are inherently inclined to explore new objects. This method has the characteristics of allowing mice to perform learning and memory tests in a free state, and can more closely simulate human learning and memory behavior. In this article, the learning and memory abilities of mice were tested by recording the exploration time of experimental mice for new objects. An established protocol was used to assess each mouse using the new object recognition (NOR) test. Mice were first placed in an arena containing two identical objects (green cube) and allowed to explore the area for 10 min. The next day, a novel object (red taper) and green cube were placed in the box, and the mice were allowed to explore for 10 min while being recorded using a camera (SMART, Panlab Harvard Apparatus, Bioscience Company, Holliston, MA, USA). We recorded the total time of exploring the novel objects. The time to cross the beam of the test was counted offline by an observer blinded to the animal treatments.

Morris water maze test

Morris water maze (MWM) experiment is to force mice to swim and learn to find the platform hidden in the water. It is mainly used to test the learning and memory ability of the experimental

animals on the sense of spatial position and direction. In this article, the learning and memory abilities of mice were evaluated by monitoring the trajectory of mice in the water maze and the time of reaching the platform. The MWM test was performed as previously described, with modifications. The apparatus used for the MWM test consisted of a water-filled circular tank. The water was made opaque by adding nontoxic white ink. During training, a hidden platform (10 cm in diameter) was placed 1 cm below the water surface in one quadrant of the tank. The mice were trained to memorize the position of the platform. If the animals failed to find the platform, they were guided to the platform and placed on the hidden platform for 30 s. The training session continued for 5 days, and the latency time was calculated. On the sixth day, the platform was removed, and a probe test was conducted. In the probe trial, the number of crossings, latency to the platform, and time spent in the target quadrant were recorded. Data were recorded using a video tracking system (SMART, Panlab Harvard Apparatus, Bioscience Company, Holliston, MA, USA).

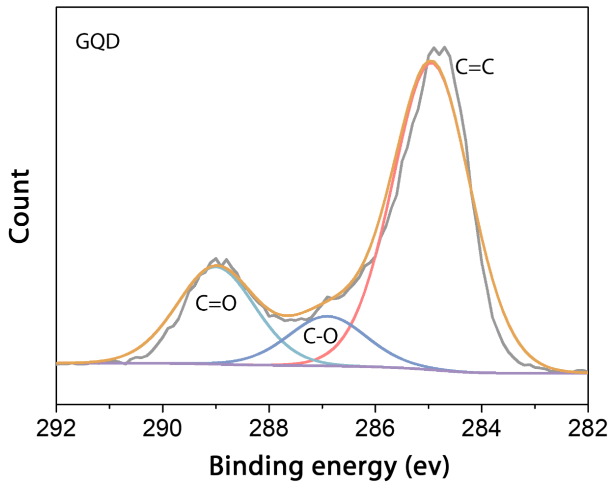

Fig. S1. High-resolution C1s X-ray photoelectron spectroscopy (XPS) data for GQD.

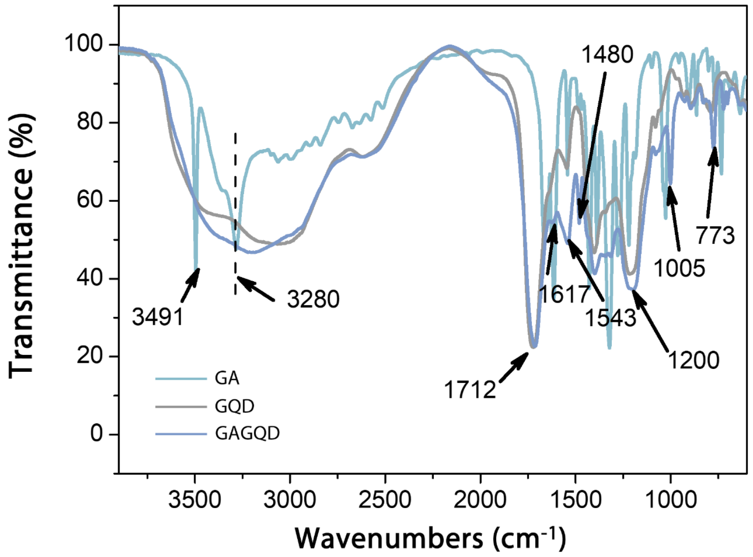

Fig. S2. Fourier transform infrared spectra of GA, GQD, and GAGQD

The spectrum of GA shows obvious peaks at 3491 cm^{-1} and 3280 cm^{-1} that are contributed by –OH stretching vibration. The FTIR spectra of GAGQD and GQD demonstrate a peak at 1712 cm^{-1} that is attributed to the stretching of the carbonyl group in the ester bond, suggesting the presence of ester groups in GAGQD and GQD. The peaks at 1617 cm^{-1}, 1543 cm^{-1} and 1480 cm^{-1} are assigned to stretching vibrations of C–C bonds in the aromatic skeleton. The peak at 1200 cm^{-1} can be indexed to the alkoxy C–O–C stretching. By comparing with GQD, the wide bands in the region of 3280 cm^{-1} in GAGQD, corresponding to the –OH groups in polyphenols (tensile vibration O–H), is more pronounced, indicating that the introduction of GA can endow GAGQD with plentiful phenolic hydroxyl groups.

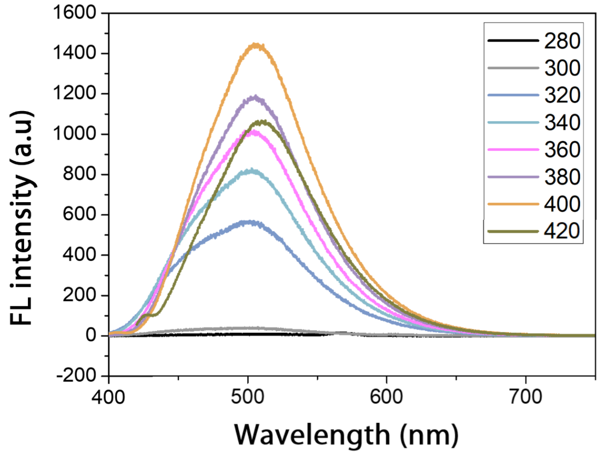

Fig. S3. Fluorescence intensity of GAGQD under 280–420 nm excitation.

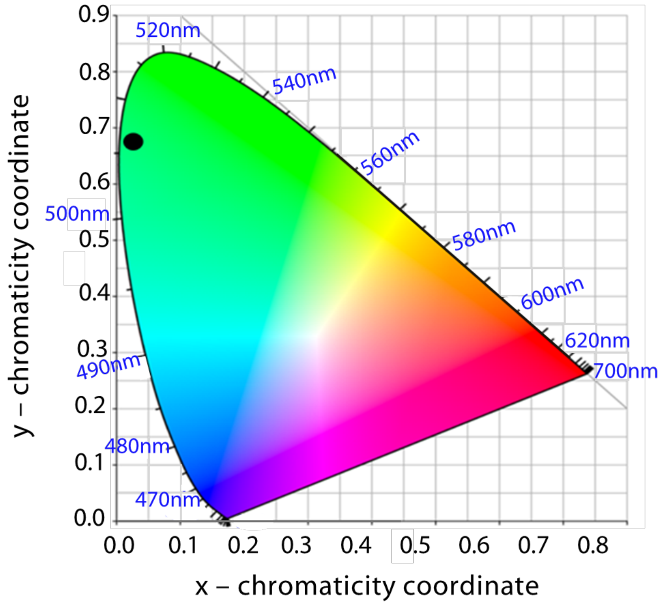

Fig. S4. CIE coordinates of GAGQD.

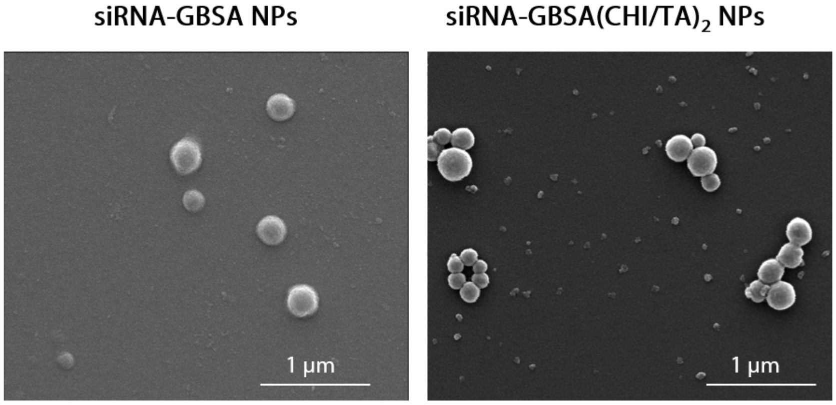

Fig. S5. SEM image of siRNA-GBSA NPs and siRNA-GBSA(CHI/TA)$_2$ NPs.

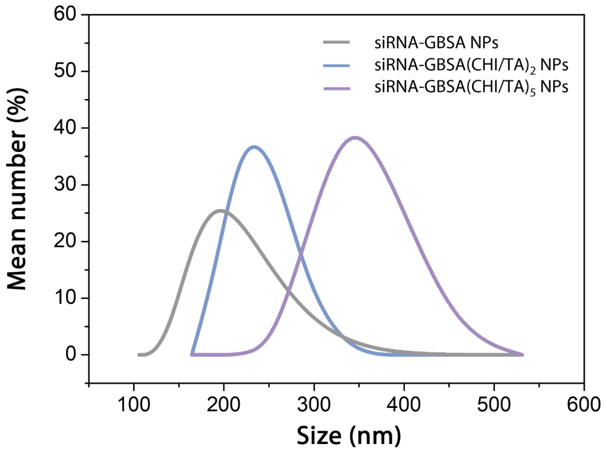

Fig. S6. Size distribution of siRNA-GBSA NPs, siRNA-GBSA(CHI/TA)$_2$ NPs, and siRNA-GBSA(CHI/TA)$_5$ NPs.

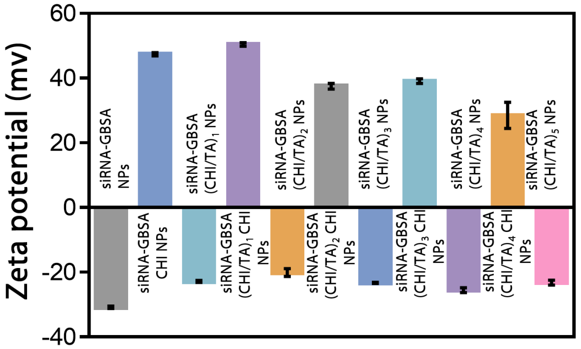

Fig. S7. Zeta potential of siRNA-GBSA NPs, siRNA-GBSACHI NPs, and siRNA-GBSA(CHI/TA)$_n$ NPs, n = 1-5.

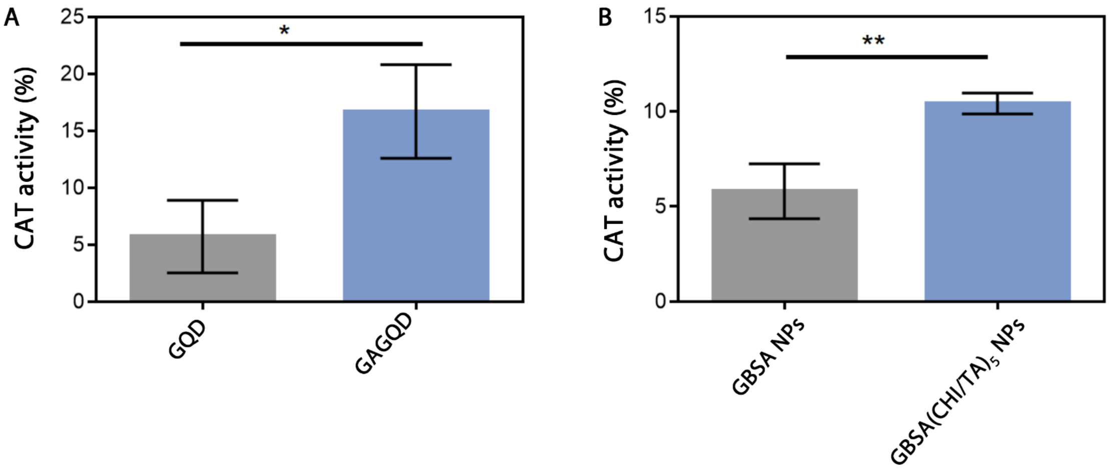

Fig. S8. The CAT activity of GQD, GAGQD, GBSA NPs and GBSA(CHI/TA)$_5$ NPs.

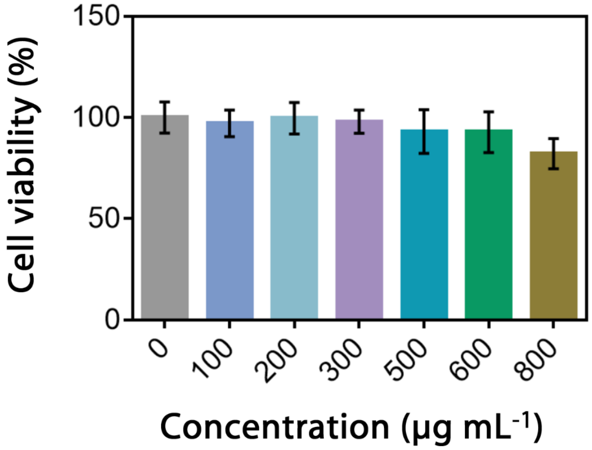

Fig. S9. Cell viability after incubation with siRNA-GBSA(CHI/TA)$_5$ NPs for 24 h. NP concentration: 0-800 μg mL^{-1}.

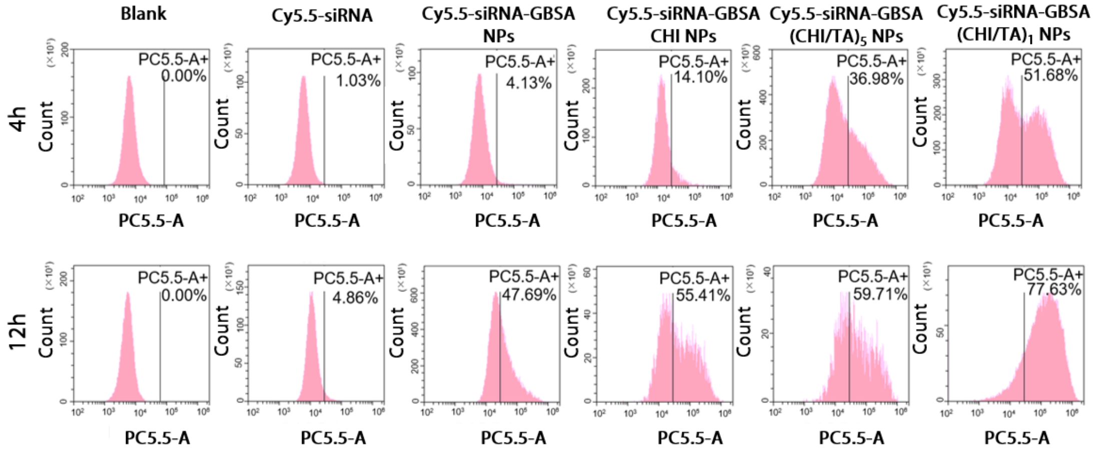

Fig. S10. Flow cytometry analysis of RAW264.7 cells after incubation with different NPs for 4 h or 12 h.

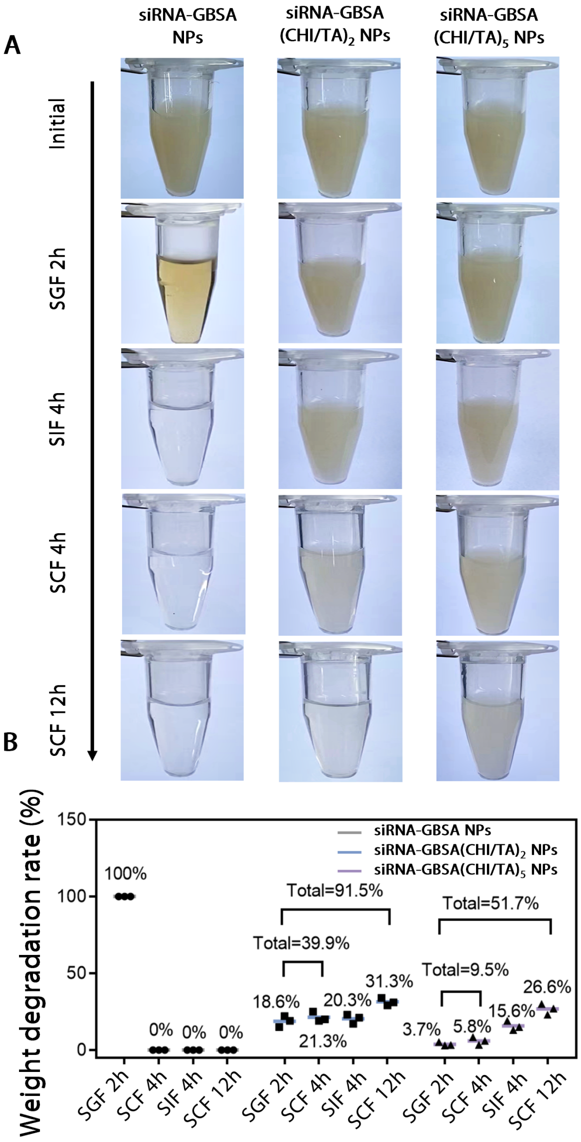

Fig. S11. (**A**) Images and (**B**) weight degradation rate of siRNA-GBSA NPs, siRNA-GBSA(CHI/TA)$_2$ NPs, and siRNA-GBSA(CHI/TA)$_5$ NPs after incubation in simulated gastric fluid (SGF) for 2 h, simulated intestinal fluid (SIF) for 4 h, and simulated colon fluid (SCF) for 4 h and 12 h.

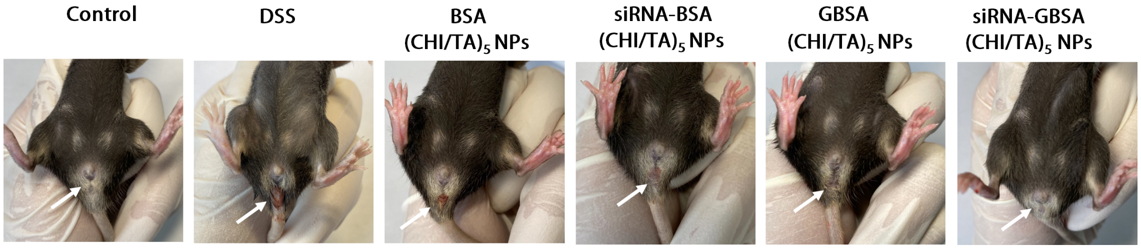

Fig. S12. Representative photographs of rectal areas of a healthy mouse and IBD mice after treatment with NPs (on the eighth day).

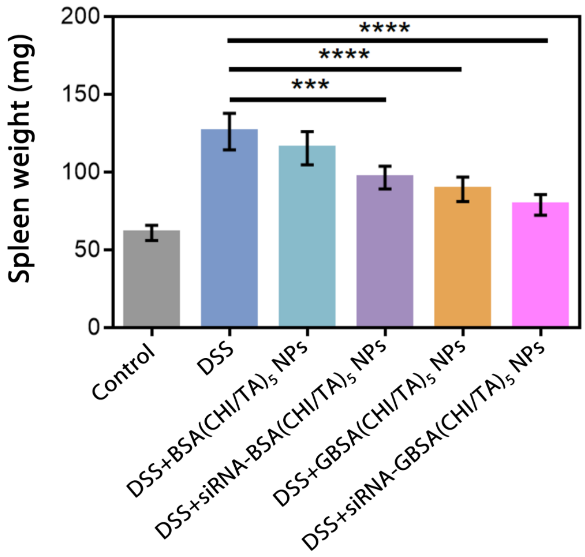

Fig. S13. Spleen weight of IBD mice after gavage administration of NPs on days 2–7, $n = 5$.

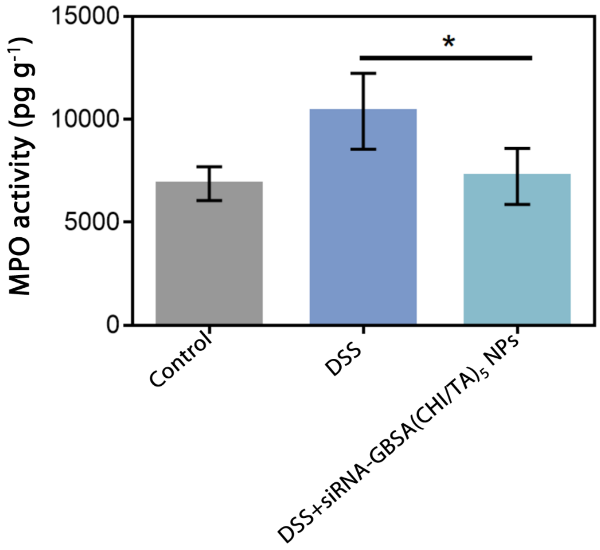

Fig. S14. MPO activity of IBD mice after gavage administration of siRNA-GBSA(CHI/TA)$_5$ NPs on days 2–7, n = 5.

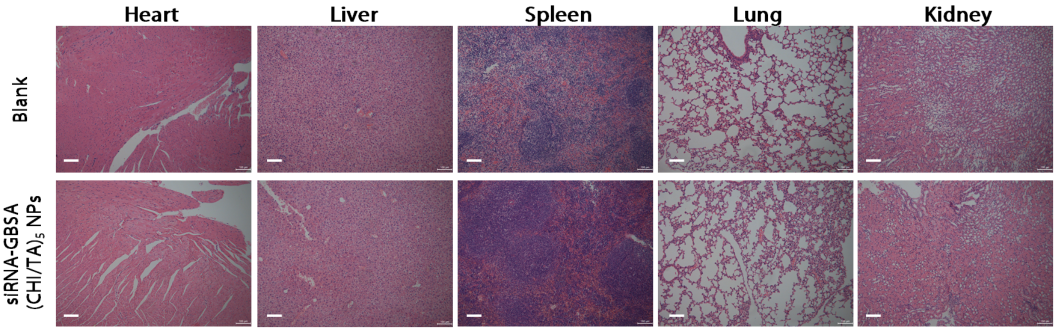

Fig. S15. H&E staining images of heart, liver, spleen, lung, and kidney tissues of mice after gavage administration of siRNA-GBSA$(CHI/TA)_5$ NPs for 6 d. Scale bar = 100 μm.

H&E staining of the heart, lung, liver, kidney, and other major organs of the mice gavage-treated with siRNA-GBSA$(CHI/TA)_5$ NPs (1 mg mL^{-1}, 200 μL day^{-1}) for 6 days was normal (same as that of the normal group of mice) (Fig. S14). These results indicate that the siRNA-GBSA$(CHI/TA)_5$ NPs exhibit good biological compatibility.

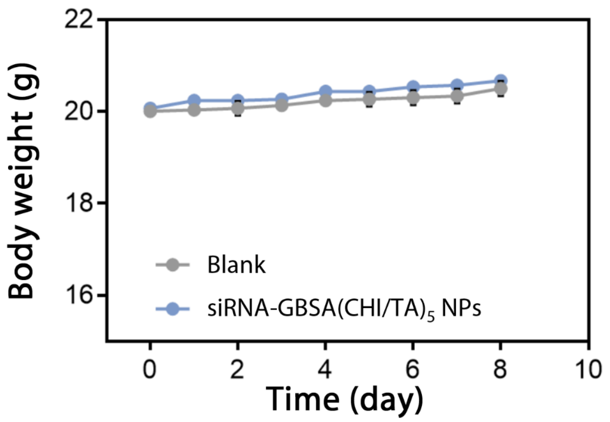

Fig. S16. Body weight records of IBD mice after gavage administration of siRNA-GBSA(CHI/TA)$_5$ NPs on days 2–7, n = 3.

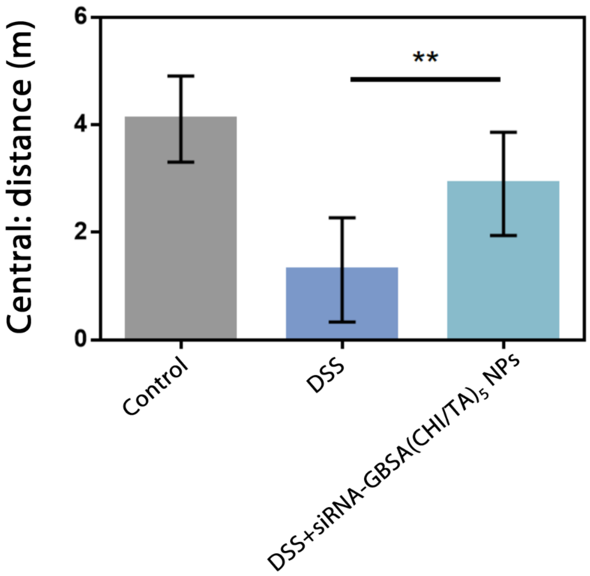

Fig. S17. Central distance of Control, DSS, and DSS+siRNA-GBSA(CHI/TA)$_5$ NPs group in the open field test, n = 10.

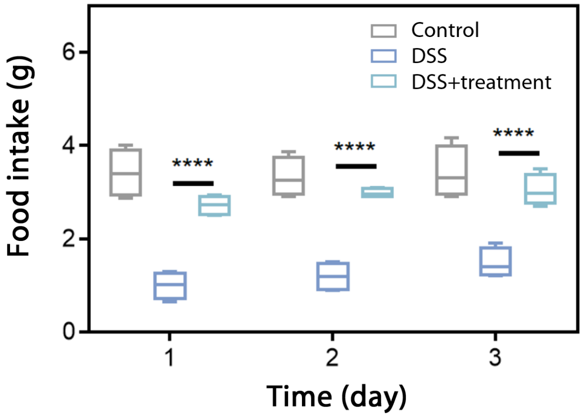

Fig. S18. Food intake of IBD mice in three days after gavage administration of siRNA-GBSA(CHI/TA)$_5$ NPs on day 2-7, $n = 4$.

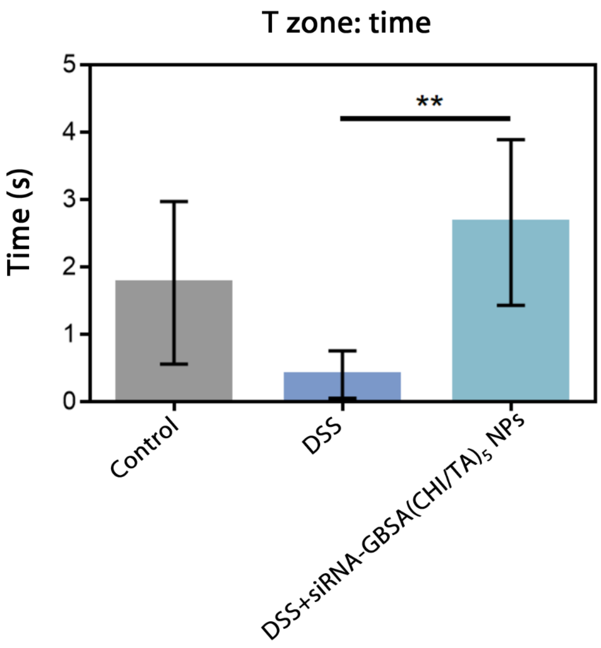

Fig. S19. Time spent of Control, DSS, and DSS+siRNA-GBSA(CHI/TA)$_5$ NPs group in the T zone in Morris water maze test, n = 10.

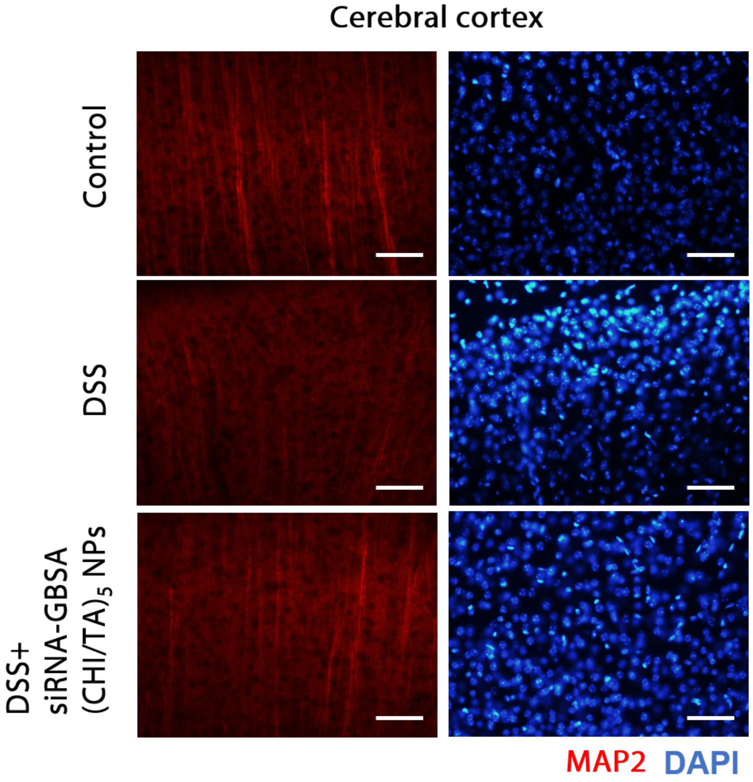

Fig. S20. Immunofluorescence staining of MAP2 in the cerebral cortex of IBD mice after gavage administration of siRNA-GBSA(CHI/TA)$_5$ NPs on days 2–7. Scale bar = 20 μm.

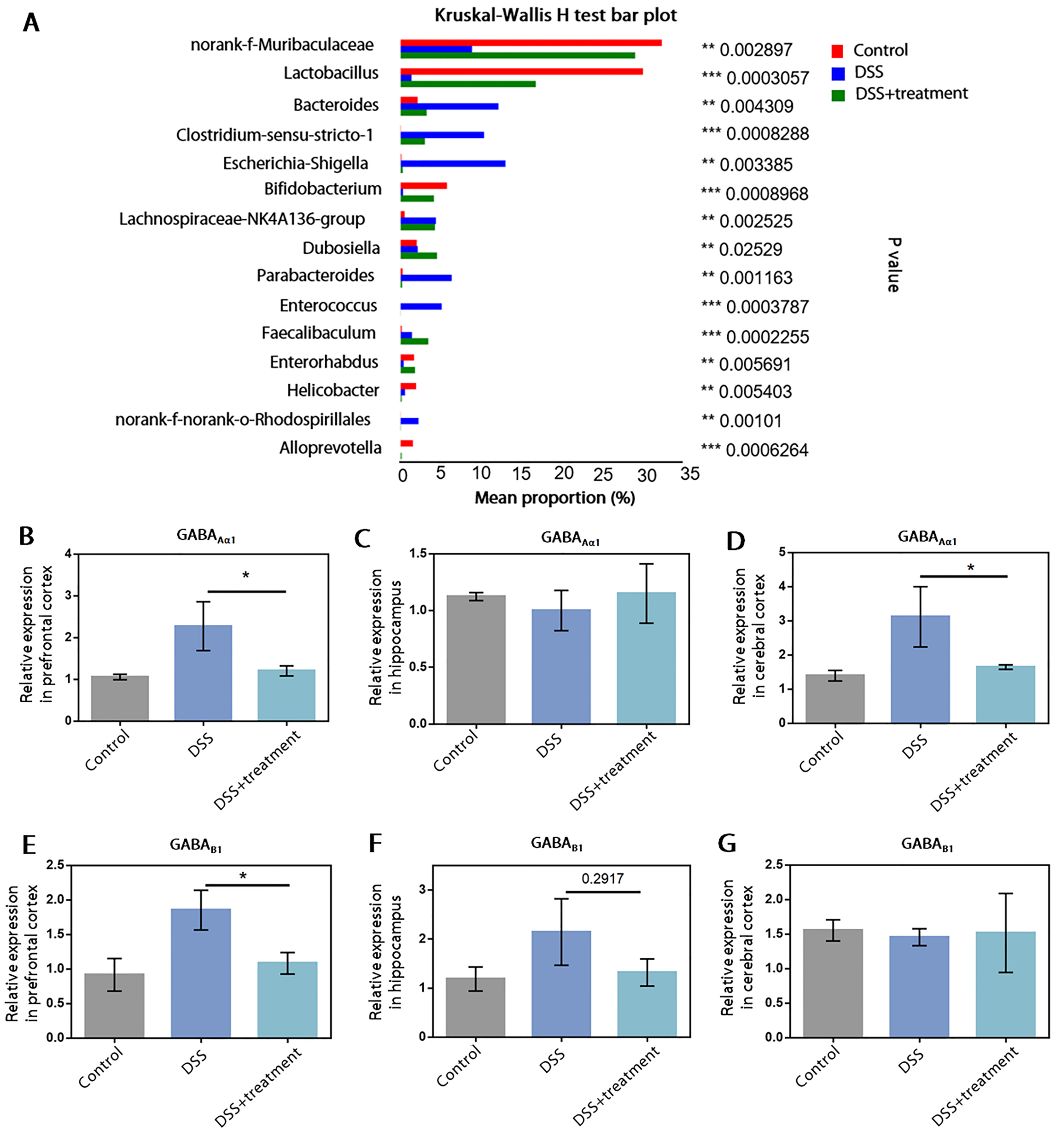

Fig. S21. (**A**) The relative abundances of flora at the genus level, each column represents the mean of each group, $n = 7$. (**B** to **D**) $GABA_{A\alpha1}$ and (**E** to **G**) $GABA_{B1}$ mRNA expression level in prefrontal cortex, hippocampus and cerebral cortex of IBD mice after gavage administration of siRNA-GBSA(CHI/TA)$_5$ NPs on day 2-7, $n = 5$.
